# Supplementary material for: Great ape cognition is structured by stable cognitive abilities and predicted by developmental conditions
Source: Nat Ecol Evol. 2023 Apr 27;7(6):927–38. doi: 10.1038/s41559-023-02050-8 (PMC10250201; doi:10.1038/s41559-023-02050-8)
Supplement: Supplementary file 1 — Supplementary Figs. 1–42, Methods and Results, and Tables 1–7. [file 41559_2023_2050_MOESM1_ESM.pdf]

nature ecology & evolution

Article

https://doi.org/10.1038/s41559-023-02050-8

Great ape cognition is structured by stable cognitive abilities and predicted by developmental conditions

In the format provided by the authors and unedited

Contents

Overview **1**

Methods **2**

Participants . . . 2

Setup . . . 2

Tasks . . . 2

Data collection . . . 5

Predictors . . . 6

Analytical framework **10**

Structural equation modeling . . . 10

Projection predictive inference . . . 15

Results **16**

Robustness, Stability and Reliability . . . 16

Relations between tasks . . . 29

Predictability . . . 33

Summary **42**

Supplementary Note **43**

Analysis and results . . . 43

Simulations . . . 46

Supplementary References **52**

Overview

This document supplements the paper "Great ape cognition is structured by stable cognitive abilities and predicted by developmental conditions". First, we give an overview of our great ape participants. Next we describe the general setup and the experimental tasks that were used. In the section data collection we lay out the time line of data collection. Next, we give an overview of the predictor variables we recorded in addition to the experimental data.

We then move on to describe the two parts of our analytical framework: Structural Equation Modeling to investigate robustness, stability and reliability of cognitive performance and Projection Predictive Inference to test the importance of the predictor variables.

We present the results separate for the two phases of data collection. For each phase, we first report results on robustness, stability and reliability of performance within each task and then we investigate relations between performance in the different tasks. Finally, we report how the different predictors related to performance in the different tasks.

The Supplementary Note contains results from simulation studies we conducted to investigate the performance of the employed Structural Equation Models under the sample sizes given in the present dataset.

Methods

Participants

A total of 43 great apes participated at least once in one of the tasks. This included 8 Bonobos (3 females, age 7.3 to 39), 24 Chimpanzees (18 females, age 2.6 to 55.9), 6 Gorillas (4 females, age 2.7 to 22.6), and 6 Orangutans (4 females, age 17 to 41.2). The sample size at the different time points ranged from 3 to 18 for the different species. Figure 1 visualizes the sample size across time points. We tried to test all apes at all time points but this was not always possible due to a lack of motivation or construction works. All apes participated in cognitive research on a regular basis. Many of them had ample experience with the very tasks we used in the current study.

Apes were housed at the Wolfgang Köhler Primate Research Center located in Zoo Leipzig, Germany. They lived in groups, with one group per species and two chimpanzee groups (group A and B). Research was noninvasive and strictly adhered to the legal requirements in Germany. Animal husbandry and research complied with the European Association of Zoos and Aquaria Minimum Standards for the Accommodation and Care of Animals in Zoos and Aquaria as well as the World Association of Zoos and Aquariums Ethical Guidelines for the Conduct of Research on Animals by Zoos and Aquariums. Participation was voluntary, all food was given in addition to the daily diet, and water was available ad libitum throughout the study. The study was approved by an internal ethics committee at the Max Planck Institute for Evolutionary Anthropology.

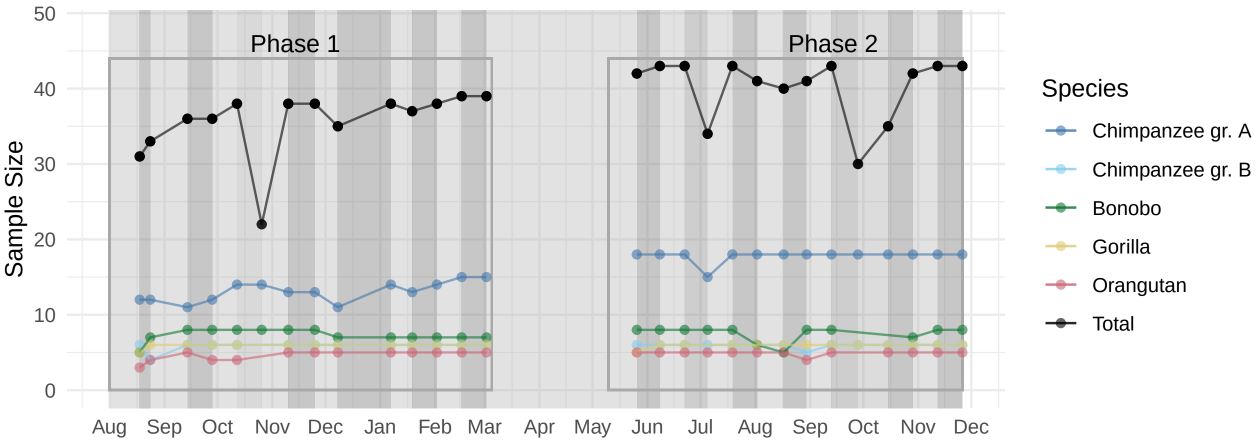

Supplementary Figure 1: Sample size by species across the different time points. Time point specific predictor variables were collected during the time between two time points (shaded regions) to predict the next.

Setup

Apes were tested in familiar sleeping or observation rooms by a single experimenter. Whenever possible, they were tested individually. The basic setup comprised a sliding table positioned in front of a clear Plexiglas panel with three holes in it. The experimenter sat on a small stool and used an occluder to cover the sliding table (see Figure 2).

Tasks

The tasks we selected are based on published procedures and are commonly used in the field of comparative psychology. The original publications often include control conditions to rule out alternative, cognitively less demanding ways to solve the tasks. We did not include such controls here and only ran the experimental

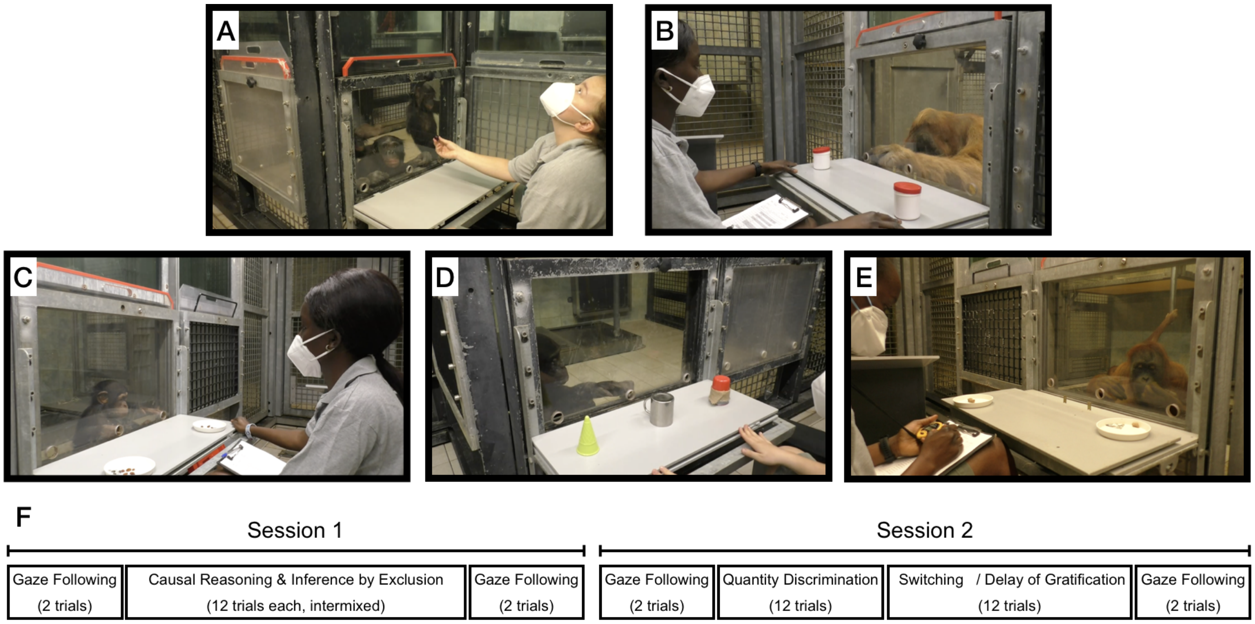

Supplementary Figure 2: Setup used for the six tasks. A) Gaze following. B) Causal reasoning and inference by exclusion. C) Quantity discrimination. D) Switching. E) Delay of gratification. F) Order of task presentation and trial numbers

conditions. For each task, we refer to the publication we used to model our procedure. We ask the reader to read these papers if they want to know more about control conditions and/or a detailed discussion of the nature of the underlying cognitive mechanisms.

Example videos for each task can be found in the associated online repository in `videos/`.

Gaze Following

The gaze following task was modeled after Bräuer, Call, & Tomasello (2005). The experimenter sat opposite the ape and handed over food at a constant pace. That is, the experimenter picked up a piece of food, briefly held it out in front of her face and then handed it over to the participant. After a predetermined (but varying) number of food items had been handed over, the experimenter again picked up a food item, held it in front of her face and then looked up (i.e., moving her head up - see Figure 2C). The experimenter looked to the ceiling, no object of particular interest was placed there. After 10s, the experimenter looked down again, handed over the food and the trial ended. We coded whether the participant looked up during the 10s interval. Apes received eight gaze following trials.

We assume that participants look up because they assume that the experimenter's attention is focused on a potentially noteworthy object.

Direct causal inference

The direct causal inference task was modeled after Call (2004). Two identical cups with a lid were placed left and right on the table (Figure 2A). The experimenter covered the table with the occluder, retrieved a piece of food, showed it to the ape, and hid it in one the cups outside the participant's view. Next, the experimenter removed the occluder, picked up the baited cup and shook it three times, which produced a rattling sound. Next, the cup was put back in place, the sliding table pushed forwards, and the participant

made a choice by pointing to one of the cups. If they picked the baited cup, their choice was coded as correct, and they received the reward. If they chose the empty cup, they did not. Participants received 12 trials. The location of the food was counterbalanced; six times in the right cup and six times in the left. Direct causal inference trials were intermixed with inference by exclusion trials (see below).

We assume that apes locate the food by reasoning that the food – a solid object – causes the rattling sound and therefore must be in the shaken cup.

Inference by exclusion

Inference by exclusion trials were also modeled after Call (2004) and followed a very similar procedure compared to direct causal inference trials. After covering the two cups with the occluder, the experimenter placed the food in one of the cups and covered both with the lid. Next, they removed the occluder, picked up the empty cup and shook it three times. In contrast to the direct causal inference trials, this did not produce any sound. The experimenter then pushed the sliding table forward and the participant made a choice by pointing to one of the cups. Correct choice was coded when the baited (non-shaken) cup was chosen. If correct, the food was given to the ape. There were 12 inference by exclusion trials, intermixed with direct causal inference trials. The order was counterbalanced: six times the left cup was baited, six times the right.

We assume that apes reason that the absence of a sound suggests that the shaken cup is empty. Because they saw a piece of food being hidden, they exclude the empty cup and infer that the food is more likely to be in the non-shaken cup.

Quantity discrimination

For this task, we followed the general procedure of Hanus & Call (2007). Two small plates were presented left and right on the table (see Supplementary Figure 2B). The experimenter covered the plates with the occluder and placed five small food pieces on one plate and seven on the other. Then they pushed the sliding table forwards, and the participant made a choice. We coded as correct when the subject chose the plate with the larger quantity. Participants always received the food from the plate they chose. There were 12 trials, six with the larger quantity on the right and six on the left (order counterbalanced).

We assume that apes identify the larger of the two food amounts based on discrete quantity estimation.

Switching

This task was modeled after Haun, Call, Janzen, & Levinson (2006). Three differently looking cups (silver metal cup with handle, green plastic ice cone, red cup without handle - Supplementary Figure 2D) were placed next to each other on the table. There were two conditions. In the place condition, the experimenter hid a piece of food under one of the cups in full view of the participant. Next, the cups were covered by the occluder and the experimenter switched the position of two cups, while the reward remained in the same location. Next, the experimenter removed the occluder and pushed the table forward. We coded as correct if the participant chose the location where the food was hidden. Participants received four trials in this condition.

The place condition was run first. The subsequent feature condition followed the same procedure, but now the experimenter also moved the reward when switching the cups. The switch between conditions happened without informing the participant in any way. A correct choice in this condition meant choosing the location to which the cup plus the food were moved. Here, participants received eight trials.

The dependent measure of interest for this task was calculated as: `[proportion correct place] - (1 - [proportion correct feature])`. Positive values in this score mean that participants could quickly switch from choosing based on location to choosing based on feature. High negative values suggest that participants did not or hardly switch strategies.

Based on the results of Haun et al. (2006), we assume that apes have a tendency to expect the food to remain in the same location. When this strategy is no longer successful in the feature trials, they have to switch strategies and try a different one.

The switching task was only used in Phase 1. It did not produce meaningful results (see results for Phase 1 in Stability and Reliability below) and for Phase 2 we therefore replaced it with a delay of gratification task (see below).

Delay of gratification

This task replaced the switching task in Phase 2. The procedure was adapted from Rosati, Stevens, Hare, & Hauser (2007). Two small plates including one and two pieces of pellet were presented left and right on the table. E moved the plate with the smaller reward forward allowing the subject to choose immediately, while the plate with the larger reward was moved forward after a delay of 20 seconds. We coded whether the subject selected the larger delayed reward (correct choice) or the smaller immediate reward (incorrect choice) as well as the waiting time in cases where the immediate reward was chosen. Subjects received 12 trials, with the side on which the immediate reward was presented counterbalanced.

We assume that, in order to choose the larger reward, apes inhibit choosing the immediate smaller reward.

Reliability coding

A second coder unfamiliar to the purpose of the study coded 15% of all time points (four out of 28) for all tasks (except switching). Reliability was good to excellent. Gaze following: 92% agreement (κ = .64), direct causal inference 99% agreement (κ = .98), inference by exclusion: 99% agreement (κ = .99), quantity discrimination: 99% agreement (κ = .97), delay of gratification: 98% agreement (κ = .97).

Data collection

One time point meant running all tasks with all participants. Within each time point, the tasks were organized in two sessions (see Supplementary Figure 2F). Session 1 started with two gaze following trials. Next was a pseudo randomized mix of direct causal inference and inference by exclusion trials with 12 trials per task, but no more than two trials of the same task in a row. At the end of Session 1, there were again two gaze following trials. Session 2 also started with two gaze following trials, followed by quantity discrimination and switching (Phase 1) or Delay of Gratification (Phase 2). Finally, there were again two gaze following trials. By spreading out or mixing tasks we hoped to keep subjects more attentive and engaged.

The order of tasks was the same for all subjects. So was the positioning of food items within each task. The counterbalancing can be found in the coding sheets in the online repository in `documentation/`. This exact procedure was repeated at each time point so that the results would be comparable across participants and time points. The two sessions were usually spread out across two adjacent days. For the larger chimpanzee group, they were sometimes spread out across four days.

The interval between two time points was planned to be two weeks. However, it was not always possible to follow this schedule so that some intervals were longer or shorter. Supplementary Figure 1 visualizes the intervals between time points.

We collected data in two phases. Phase 1 started on August 1st, 2020, lasted until March 5th, 2021 and included 14 time points. Phase 2 started on May 26th, 2021 and lasted until December 4th, 2021 and also had 14 time points (see Supplementary Figure 1).

Predictors

In addition to the data from the cognitive tasks, we collected data for a range of predictor variables. The goal here was to find variables that are systematically related to inter- and/or intra-individual variation in cognitive performance. That is, we were interested to see which variables allow us to predict cognitive performance. The second part of the analysis section describes the method we used to determine the predictive value of each variable.

Predictors could either vary with the individual (stable individual characteristics; e.g. sex or rearing history), vary with individual and time point (variable individual characteristics; e.g. sickness or sociality), vary with group membership (group life; e.g. time spent outdoors or disturbances) or vary with the testing arrangements and thus with individual, time point and session (testing arrangements; e.g. presence of an observer or participation in other tests).

Most predictors were collected via a diary that the animal caretakers filled out on a daily basis. Here, the caretakers were asked a range of questions about the presence of a predictor and its severity. The diary (in German) can be found in `documentation/` in the associated online repository.

Stable individual characteristics

These predictors are stable individual differences. As a source, we used the ape handbook at Zoo Leipzig. Supplementary Figure 3 gives an overview of the distribution of the different characteristics in the sample.

Group Group the individual belonged to. Groups were composed of individuals of the same species but because there were two chimpanzee groups (A-chimpanzees and B-chimpanzees), group and species are not equivalent. Variable name in model: `group`.

Age Absolute age of the individual. For some older individuals, only the year of birth was known. In these cases we calculated age with January 1st of that year as the birthday. Variable name in model: `age`.

Sex Participant's biological sex. Variable name in model: `sex`.

Rearing history Here, we differentiated between, `mother-reared`, `hand-reared` and `unknown`. The last category was used only for three chimpanzees. In the analysis, we classified them as `hand-reared` to facilitate model fitting (i.e. it is very difficult to estimate a parameter for a factor level with so little data). We think this decision is justified because the individuals in question have spent most of their life in close contact to humans and not in a larger chimpanzee group. Variable name in model: `rearing`.

Time spent in research Absolute time the individual has lived in Leipzig Zoo. All apes living in Leipzig are involved in behavioral research to a certain degree. Thus, we take this measure to be a rough proxy of how much experience an individual has had with cognitive research. Variable name in model: `time_in_leipzig`.

Variable individual characteristics

These predictors varied by participant and time point.

Rank We asked caretakers to order individuals within a given group according to their rank. Ties were allowed. This was done at each time point. An individual's rank was mostly stable (see Supplementary Figure 4A) across time points, however, there was some variation. Variable name in model: `rel_rank`.

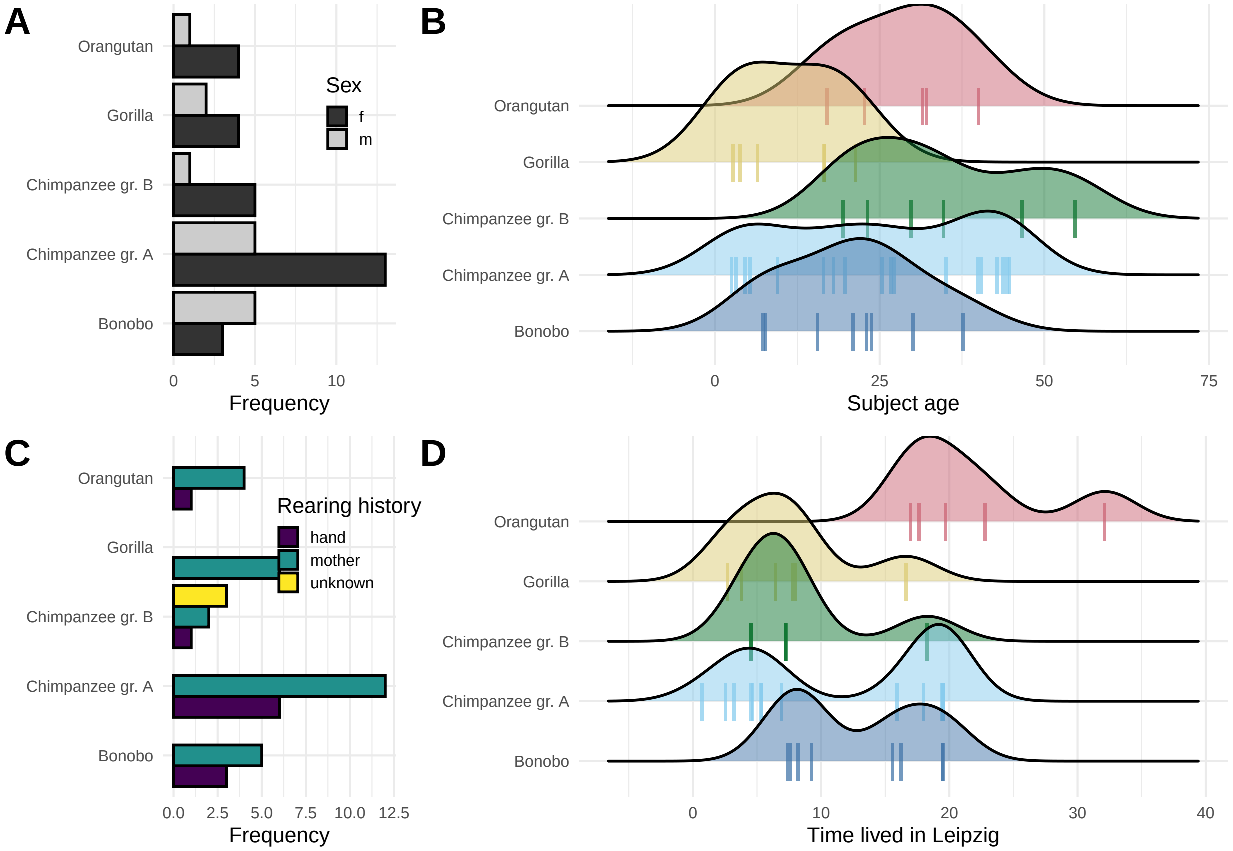

Supplementary Figure 3: Stable individual characteristics. A) participant sex, B) age distribution by species, C) rearing history, D) time lived in leipzig by species.

Sickness As part of the caretakers' daily diary, we asked whether an individual was sick and if yes, how severe the sickness was on a scale from 1 to 7. For each time point, we used the mean of the daily sickness ratings as predictor. Variable name in model: `sick_severity`.

Sociality We conducted proximity scans for all groups in the early afternoon on every workday (Monday to Friday). That is, we expect 10 scans for each time point. For each individual, we recorded which individuals were within arms reach. Research assistants used a tablet to record their observations with the behavioral coding software ZooMonitor (Wark et al., 2019).

To derive individual specific estimates of sociality for each time point, we fit a variant of a Social Relations Model (Snijders & Kenny, 1999) to the proximity data. These models allow estimating an individual specific sociality index while accounting for the dyadic nature of social interaction. Social relations models usually deal with directed behaviors (e.g. individual i is grooming individual j). Because the behavior we observed was symmetric, we cannot differentiate between the actor and receiver. @Kajokaite2020.08.04.235788 suggested to speak of a Multiple Membership Relations Model (see also Leckie, 2019) in such a context, which simply estimates how likely an individual is to be observed in proximity to another individual.

In `brms` syntax, our model had the following structure: `count | trials(n) ~ group + (time_point | mm(focal, associates)) + (time_point | dyad)`. The dependent variable `count | trials(n)` is the number of times a dyad has been observed (`count`) at a time point relative to the number of scans taken for that time point (`trials(n)`). The fixed effect `group` estimates group difference in sociality. The random effect `(time_point | mm(focal, associates))` estimates the sociality for each individual. In that, the multi-membership grouping term `mm(focal, associates)` captures the fact that the assignment of the two roles (focal and associate) is arbitrary in the context of a symmetric behavior. The random slope `time_point` (treated as a factor) allowed us to estimate sociality for each time point. Finally, the random effect `(time_point | dyad)` accounts for dyad composition; in some cases a particular dyad composition (e.g. mother and infant) might be sufficient to explain high levels of sociality in an individual.

For each individual and time point, we extracted the sociality estimates and used them to predict cognitive performance in the different tasks for that time point. Supplementary Figure 4B visualizes the sociality measures for one group across the different time points. Variable name in model: `sociality`.

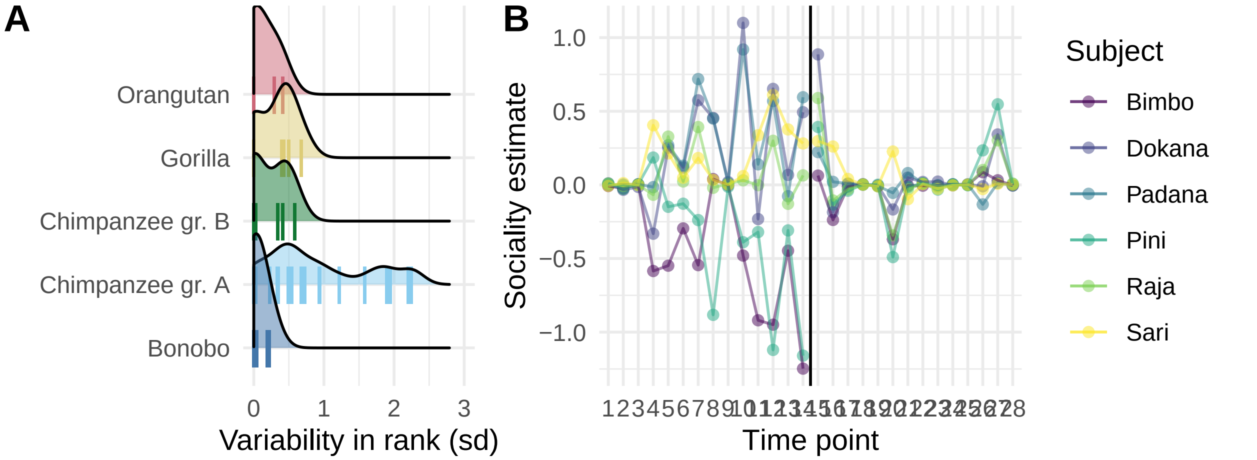

Supplementary Figure 4: Variable individual characteristics. A) variability in rank (caretaker ratings) for each subject and species, B) sociality estimates for orangutans based on Multiple Membership Relations Model.

Group life

These predictors varied by time point and group, but were the same for all individuals in that group. They were recorded in the animal caretaker diary. Supplementary Figure 5 visualizes the different variables across time points.

Time outdoors Each day, the animal caretakers noted in the diary how many hours each group spent in the outdoor enclosure instead of the indoor enclosure or the sleeping rooms. To compute the predictor, we averaged across these values for each time point and group. Variable name in model: `time_outdoors`.

Disturbances The animal caretakers also noted if there were any unusual disturbances for a particular group. Examples were construction works in the building, heavy weather conditions or green-keeping activities. In addition, the caretakers rated how disturbing they judged these events to be on a scale from 1 to 7. For each time point, we calculated the mean of these ratings. Variable name in model: `dist_mean`.

Life events This variable captured whether there were any notable events within the group. Examples were fights in the group or the temporal removal of some individuals for medical procedures. Again, we asked the caretakers to rate the severity of these events on a scale from 1 to 7 and averaged across them. Variable name in model: `le_mean`.

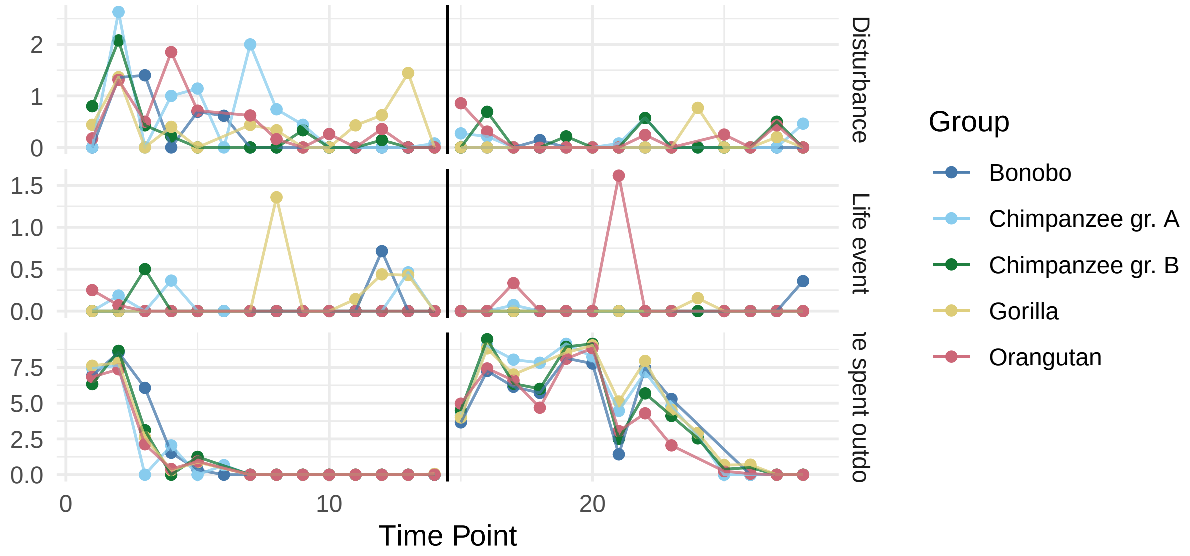

Supplementary Figure 5: Variation in group life related measures across groups and time points.

Testing arrangements

Testing arrangements varied between individuals, sessions and time points. The experimenter recorded them either based on their observations during testing or from the testing schedule, which lists all studies along with their participants that take place on a particular day.

Observer We noted whether or not there was another animal in the same room or the room adjacent to the one the participant was in. Variable name in model: `observer`.

Study on same day This predictor recorded whether or not the participant had already participated in a different study on the same day. The experimenter took this information from the testing schedule. Variable name in model: `test_day`.

Studies since last time point Here we counted in how many other studies the participant had taken part in since the last time they were tested in that particular task. The experimenter took this information from the testing schedule. Variable name in model: `test_tp`.

Analytical framework

We had two overarching questions. On the one hand, we were interested in the cognitive measures and the relations between them. That is, we asked how robust performance in a given task was on a task-level, how stable individual differences were and how reliable the measures were. We also investigated relations between the different tasks. We used *Structural Equation Modeling* (SEM) (Bollen, 1989; Hoyle, 2012) to address these questions. SEMs usually require larger sample sizes than available in the present study. In the appendix we present results from a small simulation study which show that parameters in the employed SEMs are accurately estimated using Bayesian estimation techniques given our available sample sizes under reasonable model restrictions. We lay out the restrictive assumptions we imposed on the parameters in the text below.

Our second question was, which predictors explain variability in cognitive performance. Here we wanted to see which of the predictors we recorded were most important to predict performance over time. This is a variable selection problem (selecting a subset of variables from a larger pool) and we used *Projection Predictive Inference* for this (Piironen, Paasiniemi, & Vehtari, 2020).

Structural equation modeling

In the present analyses we were interested in estimating the stability of performances in a given task across time as well as the association between performances across different tasks. To separate components of random fluctuation (measurement error) from systematic differences in performance across time, we used Structural equation models (SEM). SEMs can be used to model relations between latent variables (constructs) which are estimated based on several observed variables.

We used latent state-trait models to separate traits (stable over time) from state residuals (time varying). In the present context, one can think of a trait as a stable psychological ability (e.g. ability to make causal inferences) and state residuals as time-specific deviations from these traits due to variable psychological conditions (e.g. variations in performance due to being attentive or inattentive). Variation in performance on a given time point can then be partitioned into variance due to the trait, variance due to the situation or individual-situation interactions, and measurement error. Because the latent variables are estimated on multiple indicators (here: test halves), they are assumed to be measurement-error free (Geiser, 2020; Steyer, Ferring, & Schmitt, 1992; Steyer, Mayer, Geiser, & Cole, 2015). Next we describe the model construction process in more detail.

At each time point, we observed several identical trials per individual per task. Using the individual trials (8 to 12 dichotomous items, depending on the task) as indicators for a latent ability factor per time point (i.e., assuming a Rasch model per time point) in a longitudinal SEM resulted in estimation problems due to many empty cells in the bivariate distributions across time and / or tasks. Therefore we decided to model sum scores of the repeated trials, given that each trial per task was an identical repetition of the same task.

To separate reliable from unreliable variance components and obtain reliability estimates of the resulting sum score variables, we build two sum score variables per task per time point. That is, for each task, two parallel test halves were build, corresponding to performance sum scores of half of the trials of the same time point per task. Trials were alternately assigned to the first and the second test half. For tasks with 12

trials per time point this procedure resulted in two test halves assuming 7 possible values (0 to 6 correctly solved trials), for tasks with 8 trials per time point, test halves could maximally assume 5 possible values (0 to 4 correctly solved trials). We interpreted reliability estimates in the following way: acceptable = .7, good = .8 and high = .9. Please note that these estimates are for test-halves and the reliability of the full would be higher.

The two test halves served as indicators for a common latent construct per time point, assuming parallel test halves (i.e., factor loadings set to 1 and assuming equal reliability). Due to only few different observed values and skewed distributions of the sum score variables, indicators were modeled as ordered categorical variables, using a probit link function. The models thereby correspond to normal-ogive graded response models (Samejima, 1969, 1996). That is, the models assume a continuous latent ability underlying the discrete responses, with an increasing probability of more correctly solved trials with increasing ability.

For model parsimony, to improve estimation accuracy (see simulation studies), and in order to test for latent mean differences across time, we assume strict factorial (or measurement) invariance across time (Meredith, 1993; Millsap & Yun-Tein, 2004). That is, in each model (task), loading parameters are set to 1 at all time points, residual variances are equal to 1 (by definition of the graded response model as detailed below), and threshold parameters (see below for details) are set invariant across time points. In other words, we assume that the indicators (test halves) measure the latent variable in an equivalent and stable manner over time.

Models and coefficients

For each task, we constructed two different models which increased in complexity. We started with a latent state (LS) model, which estimates a latent state for each time point based on the two test halves. Robustness of task-level performance can be assessed by comparing latent state means across time points. Stability of individual differences can be assessed by correlating latent state variables across different time points.

Second, we fit a latent state-trait (LST) model. In LST models, true inter-individual differences are decomposed into a latent trait variable and time-specific deviations of the true score from the latent trait (state residual variable). In the following LST models, we assume stable latent trait variables across time (no trait change). The model allows us to partition the true variance in performance into stable (trait) and variable (state residual) components. Assuming a stable latent trait variable, the LST model is more restrictive than the LS model with respect to the implied covariance matrix, as correlations between true scores are not freely estimated across time points but assumed to be the same for different time lags.

In addition, we also fit an LST model with autoregressive effects (LST-AR). In addition to the LST model architecture, this model assumes that the state residual variables at one time point can be used to predict the true score at the subsequent time point. As such, it captures the idea that measurements that are closer in time are likely to be more highly correlated and quantifies potential carry-over effects from one time point to the next. In the present context, however, this class of models proofed difficult to interpret. Whenever critical parameters obtained reasonable values, the interpretation was in line with the regular LST models. In most cases, however, some model parameters suggest that the models are not empirically identified. We therefore do not base our conclusions on these models. Nevertheless, we provide a detailed description in the appendix.

The following sections give a mathematical description of the different models and the parameters in them.

Latent state models In the following we chose a factor analytical representation of the graded response model, that is, we present the models as factor models for ordinal data. Thereby we assume that the observed categorical variables Y_{it} for test half i at time point t result from a categorization of unobserved continuous latent variables Y^*_{it} which underlie the observed categorical variables. For observed variables that take on k_{it} different ordered values out of the set of possible categories $S_{it} = 0, ..., k_{it} - 1$ the relation between Y_{it} and Y^*_{it} is described by:

$$Y_{it} = \begin{cases} 0 & for \quad Y^*_{it} \leq \kappa_{1it} \\ s & for \quad \kappa_{sit} < Y^*_{it} \leq \kappa_{(s+1)it} \qquad with \;\; 0 < s < k_{it} - 1 \\ k_{it} - 1 & for \quad \kappa_{(k_{it}-1)it} < Y^*_{it} \end{cases} \tag{1}$$

where κ_{sit} denote threshold parameters (B. Muthén, 1984).

The graded response model assumes that the different categories of responses (in our case the number of correct trials per test half) form an ordered scale. Which category an individual scores depends on their latent ability. Because the latent variable is continuous but the response is discrete, there are thresholds on the latent ability that mark the transition between response categories. The threshold parameters κ_{sit} correspond to the level of the latent ability necessary to respond in category s or higher with 0.50 probability.

In latent state models, these continuous latent variables Y^*_{it} are decomposed into a latent state variable S_t and a measurement error variable ϵ_{it} (see, for instance Eid & Kutscher, 2014):

$$Y^*_{it} = S_t + \epsilon_{it} \tag{2}$$

with $\epsilon_{it} \sim N(0,1) \; \forall \; i, t$ (probit parameterization; normal-ogive graded response model). See Takane & De Leeuw (1987) for the equivalence of the normal-ogive graded response model and the factor model with ordinal indicators.

At each time point t, the two latent variables Y^*_{1t} and Y^*_{2t} are assumed to capture a common latent state variable S_t. Latent state variables are allowed to freely correlate across time, with latent (measurement-error free) correlations serving as indirect indicators of stability across time. The model is depicted for six measurement time points in Supplementary Figure 6.

To test for possible mean changes of ability across time, the means of the latent state variables are freely estimated (assuming invariance of the threshold parameters κ_{sit} across time).

As an estimate of reliability, the proportion of true score variance relative to the total variance of the continuous latent variables Y^*_{it} is computed:

$$Rel(Y^*_{it}) = \frac{Var(S_t)}{Var(S_t) + Var(\epsilon_{it})} = \frac{Var(S_t)}{Var(S_t) + 1} \tag{3}$$

Latent state-trait (LST) models In LST models, the latent state variables S_{it} are further decomposed into a latent trait variable T_{it} and a latent state residual variable ζ_{it}. The latent trait variables T_{it} are time-specific dispositions, that is, trait scores capture the expected value of the latent state (i.e., true score) variable for an individual at time t across all possible situations the individual might experience at time t (Eid, Holtmann, Santangelo, & Ebner-Priemer, 2017; Steyer et al., 2015). The state residual variables ζ_{it} capture the deviation of a momentary state from the time-specific disposition T_{it}. In the following models, we assume that latent traits are stable across time. Additionally assuming common latent trait and state residual variables across the two test halves, results in the following measurement equation for parcel i at time point t:

$$Y^*_{it} = T + \zeta_t + \epsilon_{it} \tag{4}$$

Here, T is a stable (time-invariant) latent trait variable, capturing stable inter-individual differences between individuals. The state residual variable ζ_t captures time-specific deviations of the respective true score from the trait variable at time t, and thereby captures deviations from the trait due to situation or person-situation interaction effects. ϵ_{it} denotes a measurement error variable, with $\epsilon_{it} \sim N(0,1) \; \forall \; i, t$. The model is depicted for 6 measurement time points in Supplementary Figure 7.

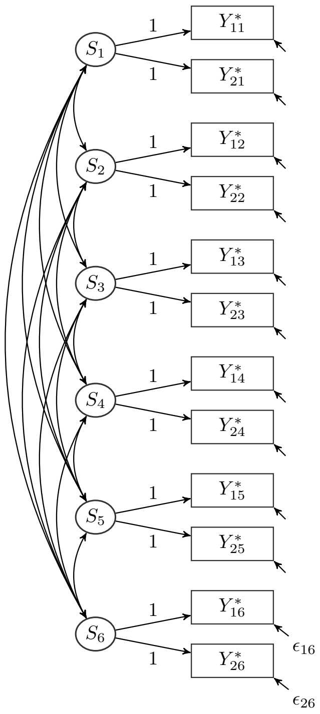

Supplementary Figure 6: Latent State model for two indicators and six measurement time points.

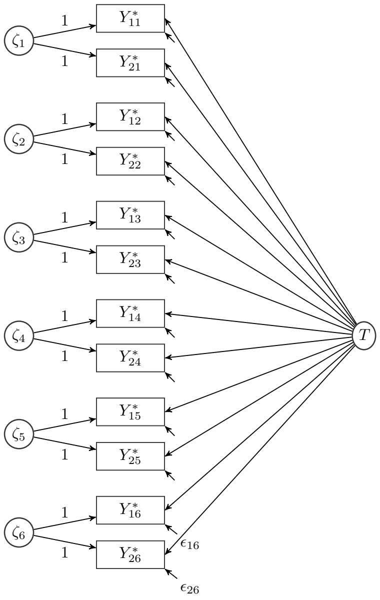

Supplementary Figure 7: Latent State-Trait model for two indicators and six measurement time points. All factor loadings of the latent trait factor T are fixed to 1 (not displayed in the figure)

As noted above, we assume strict factorial (measurement) invariance. Additionally, for reasons of parsimony, we assume that the variances of the state residual variances are invariant across time. As a consequence, the specified LST model corresponds to a multilevel model with a latent trait factor at the between-level (person-level) and a latent state residual factor at the within-level (time-specific) level.

The following variance components can be computed for the presented LST model.

Consistency Proportion of true variance (i.e., measurement-error free variance) that is due to true inter-individual stable trait differences.

$$Con(Y_{it}^*) = \frac{Var(T)}{Var(T) + Var(\zeta_t)} \tag{5}$$

Occasion specificity Proportion of true variance (i.e., measurement-error free variance) that is due to true inter-individual differences in the state residual variables (i.e., occasion-specific variation not explained by the trait).

$$OS(Y_{it}^*) = 1 - Con(Y_{it}^*) = \frac{Var(\zeta_t)}{Var(T) + Var(\zeta_t)} \tag{6}$$

As state residual variances $Var(\zeta_t)$ were set equal across time, $Con(Y_{it}^*)$ and $OS(Y_{it}^*)$ are constant across time (as well as across item parcels i).

Models for combinations of tasks To investigate associations between cognitive performance in different tasks, the described models were extended to multitrait models. Due to the small sample size, we could not combine all tasks in a single, structured model. Instead, we assessed relations between tasks in pairs.

Estimation

Models were estimated with MPlus version 8.4 (L. K. Muthén & Muthén, 1998-2017), using Bayesian Markov-Chain Monte-Carlo sampling, with the Mplus default priors (see simulation studies in the appendix). Using inverse gamma priors `IG(0.001, 0.001)` for LST models did not substantially change the parameter estimates (see simulation study). Therefore, only the results using the MPlus default priors are reported. We used two chains with a minimum of 10,000 iterations per chain, with a thinning of 10 (corresponds to a minimum of 100,000 drawn samples per chain of which every 10th is used for the construction of the posterior distribution). The first half of each chain is discarded as burn-in. Convergence was assumed and estimation stopped when the Potential Scale Reduction (PSR) factor was well below a threshold of 1.01 for the first time after the minimum number of iterations was reached. Model syntax can be accessed by locating the respective model in the folder `writing/supplement/saves/` in the GitHub repository and opening the `.out` file using a text editor.

Model fit was evaluated by computing Posterior Predicted P-values (PPP). PPP is the probability that the newly generated data are more extreme than the observed data, as measured by a specific test statistic or discrepancy function, in this case the chi-square fit function (that is, the likelihood ratio test between the specified structural equation model and an unrestricted mean and variance covariance model), see Asparouhov & Muthén (2010). The PPP is computed via the following steps: For a given MCMC iteration, a new data set is generated based on the model and the parameters of that iteration. Then the likelihood ratio chi-square test is applied to the real data as well as the newly generated data set to compute a fit index. The indices for the data and the generated data are then compared in size. If the value for the data is smaller, it is scored as 1 and if not, as 0. Averaging across these scores for the different iterations yields the PPP. Thus, values around .5 suggest a good model fit (no systematic difference between real and generated data) and very high and very low values suggest a poor model fit and / or model misspecification. In addition,

we report the 95% CI of the difference between predicted and observed chi-square values, which should be centered around 0 for a good model fit.

In Mplus, every 10th iteration after burn-in is used to compute the PPP and the underlying continuous response variables Y^* are used to compute the PPP in case of ordinal data.

Projection predictive inference

Our goal is to select the predictor variables that are relevant for predicting performance in the different cognitive tasks over time. The selection of relevant predictor variables constitutes a variable selection problem, for which a range of different methods are available (e.g., shrinkage priors). We chose to use projection predictive inference because it is a state-of-the-art variable selection procedure that provides an excellent trade-off between model complexity and accuracy (Piironen & Vehtari, 2017), especially when the goal is to identify a minimal subset of predictors that yield a good predictive model (Pavone, Piironen, Bürkner, & Vehtari, 2020).

An overview of different projection techniques and an introduction to the projection prediction approach for generalized linear models can be found in Piironen et al. (2020). In this work, we use the extension to the generalized linear multilevel model case provided by Catalina, Bürkner, & Vehtari (2020).
The projection prediction approach can be viewed as a two-step process: The first step consists of building the best predictive model possible, called the reference model. In the context of this work, the reference model is a Bayesian multilevel regression model (repeated measurements nested in apes), including all available predictors. The reference model serves as a performance goal regarding the predictive quality for the smaller models constructed by the projection prediction procedure.

In the second step, the goal is to replace the posterior distribution of the reference model with a simpler distribution. This is achieved via a forward step-wise addition of predictors that decrease the Kullback-Leibler (KL) divergence from the reference model to the projected model. Let the reference model and the projected model have parameters θ' and θ respectively. Then by the definition of the KL divergence, the following optimization problem is obtained:

$$\begin{aligned} \theta_{\perp} &= \arg\min_{\theta} \frac{1}{n} \sum_{i=1}^{n} \mathrm{KL}\left(p\left(\tilde{y}_i \mid \theta'\right) \| p\left(\tilde{y}_i \mid \theta\right)\right) \\ &= \arg\min_{\theta} \frac{1}{n} \sum_{i=1}^{n} p\left(\tilde{y}_i \mid \theta'\right) \cdot \log\left(\frac{p\left(\tilde{y}_i \mid \theta'\right)}{p\left(\tilde{y}_i \mid \theta\right)}\right) \end{aligned} \tag{7}$$

The result of the projection is a list containing the best model for each number of predictors from which the final model is selected by inspecting the mean log-predictive density (`elpd`) and root-mean-squared error (`rmse`). The projected model with the smallest number of predictors which shows similar predictive performance as the reference model is chosen.

We built separate (Bayesian multilevel regression) reference models for each task and phase and ran them through the above-described projection prediction approach. The dependent variable for each task was the cognitive performance of the apes, that is, the number of correctly solved trials per time point and task. The model for the delay of gratification task was only estimated once (Phase 2).

The reference model for the gaze following task additionally allowed for the trend over time to vary by ape (random slope for `time_point`). This additional random slope was added due to convergence issues in the random intercept model for the gaze task. While adding the random slope led to the convergence of the gaze task models, it did not fully resolve the problem for the projection. This is indicated by the form of the marginals of the projected posteriors, which still show a not entirely expected form of distribution for the gaze tasks. However, given that the model converged and the posterior distributions show only some unexpected behavior, we proceed with the analysis. Furthermore, at this point in time, the `projpred` package does not allow for fitting more complex models which might have helped to alleviate this problem.

Continuous predictors were centered when needed. We transformed the `rank` variable into a relative rank, where a rank of value one depicts a subject with the highest possible rank. All models also included `time_point` as a predictor to assess changes that are related to time and thus task experience (learning or habituation). We added the predictor `day2` for gaze following, indicating whether the trials were from the second session or the first. All reference models converged well, having no divergent transitions, R-hat values equal to 1, and large ESSs for virtually all parameters. The R-hat value is a diagnostic value to investigate the convergence of the model and refers to the same concept as the potential scale reduction (PSR) factor defined above. R-hat values close to 1 indicate that the chains have mixed well (the estimates of the chains agree with each other), while values above 1 indicate that the chains did not converge to the same value. For chains of autocorrelated samples, the effective sample size (ESS) is an estimate for the number of independent samples within a chain containing the same amount of information about the dependent variable.

Following step two, we performed projection prediction for each reference model separately, thus resulting in different rankings of the relevant predictors for each task and phase. We used the R package `projpred` (Piironen, Paasiniemi, Catalina, Weber, & Vehtari, 2022), which implements the aforementioned projection prediction technique. The predictor relevance ranking is measured by the LOO cross-validated mean log-predictive density and root-mean-squared error. To find the optimal submodel size, we inspected – in line with the authors' recommendations – summaries and the plotted trajectories of the calculated `elpd` and `rmse`.

The order of relevance for the predictors and the random intercept (together called terms) is created by performing forward search. The term that decreases the KL divergence between the reference model's predictions and the projection's predictions the most goes into the ranking first. Forward search is then repeated N times to get a more robust selection. We chose the final model by inspecting the predictive utility of each projection. To be precise, we chose the model with p terms where p depicts the number of terms at the cutoff between the term that increases the `elpd` and the term that does not increase the `elpd` by any significant amount. In order to get a useful predictor ranking, we manually delayed the random intercept term to the last position in the predictor selection process. The random intercept delay is needed because if the random intercept were not delayed, it would soak up almost all of the variance of the dependent variable before the predictors are allowed to explain some amount of the variance themselves. One could have used the function `suggest_size` as a heuristic decision rule to find the optimal submodel as an alternative to a graphical inspection. However, this is not yet possible due to the delay of the random intercept term.

Results

Robustness, Stability and Reliability

As mentioned above, we fit two different SEMs to the data from each task. Each model offers a slightly different perspective on how robust, stable and reliable performance is. We report the results starting with the LS model, followed by the LST model.

Reliability is defined as the proportion of true-score variance relative to the total (observed) variance, that is, in the context of the specified SEMs, variance that is explained by the latent state (residual) and trait variables. Reliability is estimated based on the correlations between indicators. Because the two indicators corresponded to the two test halves in our case, reliability was equivalent to a split-half reliability estimate.

In the LS models, we can look at the robustness of task-level performance by comparing the latent means estimated for each time point to see if they differ substantially from one another. To assess the stability of individual differences, we can look at the correlations between the latent state estimates for the different time points.

For LST models, we can assess stability of individual differences by looking at consistency and occasion specificity estimates. A high level of consistency means that a large portion of the variation observed in performance at the different time points can be traced back to variation in the overarching trait. High levels of occasion specificity mean the inverse, namely that large portions of the variation in performance is

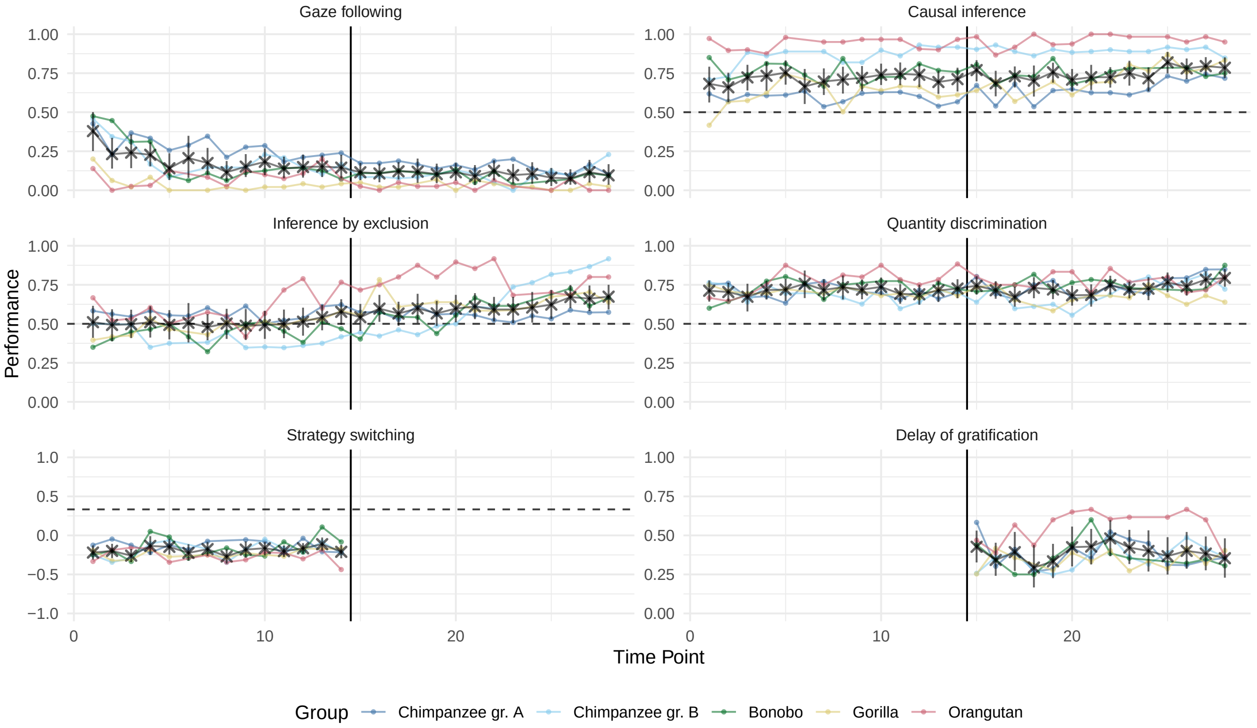

Supplementary Figure 8: Results from the five cognitive tasks across time points. Black crosses show mean performance at each time point across species (with 95% CI). The sample size varied between time points and can be found in Supplementary Figure 1. Colored dots show mean performance by species. Dashed line shows the chance level whenever applicable. The vertical back line marks the transition between phase 1 and 2.

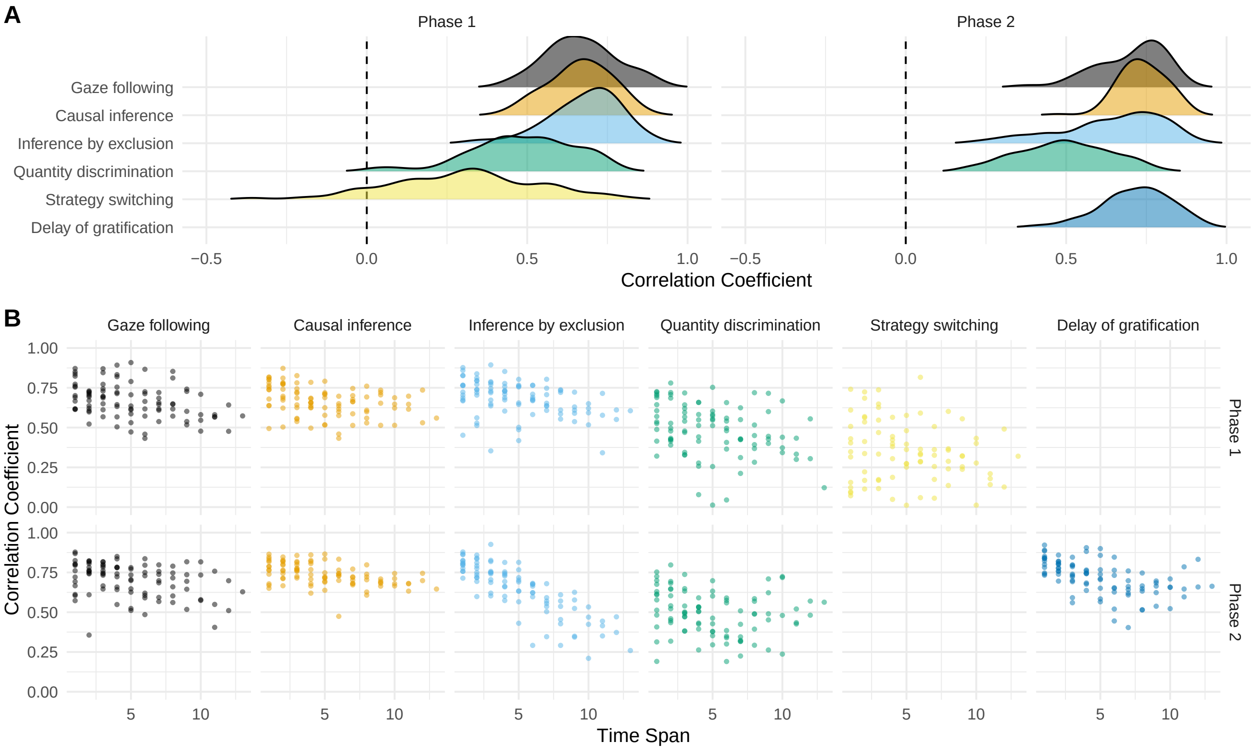

Supplementary Figure 9: (A) Distribution of correlations between time points for each task. (B) Re-test correlation coefficients plotted against the temporal distance between the testing time points.

explained by variation in the state residual – that is, occasion-specific variation not explained by the trait, due to situation and/or person-situation interaction effects.

We ran the same models for the data from Phase 1 and Phase 2. We first report the results for each task separately for the two phases and then compare how they differ between phases.

Phase 1

To get an overview of the results, we first visualized the data. Supplementary Figure 8 shows performance at the different time points. From a task-level perspective, we can say that performance was consistently above chance (0.5) in the direct causal inference and quantity discrimination tasks. For gaze following, there is no meaningful chance level. We can note, however, that task-level performance never went down to zero, which would be expected if apes did not pay attention to the experimenter's gaze. The performance score in the switching task was largely negative, suggesting no successful switching between the two phases.

For a first glimpse on the stability of individual differences, we correlated performance at the different time points for each task (all possible combinations of time points). Supplementary Figure 9A visualizes the distribution of raw correlations between the different time points and 9B plots the re-test correlation coefficients against the temporal distance between time points. Correlations between time points were large and exclusively larger than zero for direct causal inference, inference by exclusion and gaze following. For quantity discrimination, this distribution was wider and overlapped with zero, but was still mostly positive. For switching, the distribution was even wider and substantially overlapped with zero. For all tasks, correlations between time points tended to be lower for time points that were further apart (Uher, 2011). However, these re-test correlations confound measurement precision (reliability) and stability of individual differences. That is, low correlations (like e.g. for quantity discrimination) could reflect high measurement error or a lack of stability of individual differences. We tease these components apart in the SEM models reported below.

Supplementary Table 1: Model fit indices Phase 1

Task	Model	PPP	Chi 95% CI
Causal inference	LSM	0.254	-87.06 ; 148.51
	LSTM	0.224	-72.05 ; 172.45
Inference by exclusion	LSM	0.336	-88.00 ; 137.28
	LSTM	0.145	-48.42 ; 183.31
Gaze following	LSM	0.537	-124.90 ; 106.92
	LSTM	0.360	-99.09 ; 136.89
Quantity discrimination	LSM	0.485	-103.64 ; 119.70
	LSTM	0.508	-116.33 ; 117.76

Note:
LSM = Latent state model
LSTM = Latent state-trait model
PPP = Posterior predictive p-value. Cut-off values (two-tailed): 0.025 and 0.975
Chi 95% CI = 95%CI of difference between predicted and observed chi-square values

Supplementary Table 2: Threshold parameters Phase 1

Task	Model	T1	T2	T3	T4	T5	T6
Causal inference	LSM	-2.711	-1.728	-1.088	-0.092	0.905	
	LSTM	-2.892	-1.907	-1.268	-0.27	0.728	
Inference by exclusion	LSM	-2.795	-1.599	-0.715	0.628	1.444	2.672
	LSTM	-2.874	-1.652	-0.736	0.663	1.522	2.808
Gaze following	LSM	-1.276	-0.01	1.095			
	LSTM	0.086	1.402	2.547			
Quantity discrimination	LSM	-1.364	-0.752	0.356	1.411		
	LSTM	-1.398	-0.802	0.254	1.237		

Note:
LSM = Latent state model
LSTM = Latent state-trait model
T1-6 = Threshold parameters for response categories

We excluded the switching task from further analysis for two reasons. First, task-level scores were constantly negative and performance in the feature trials always overlapped with chance. This suggests that, as a group, apes did not successfully switch strategies (see Supplementary Figure 8). Second, the correlations between the different measurement time points were low, suggesting no systematic individual differences (see Supplementary Figure 9).

Next, we report the SEM results for the different tasks and the relations between them. All models showed acceptable fit indices (see Supplementary Table 1). The threshold parameters for each model are shown in Supplementary Table 2.

Direct causal inference To fit the models, the response categories of 0 or 1 solved trial had to be collapsed into one category due to sparsity. Furthermore, the thresholds could not be set equal for test-half 2 at time point 3 and 11 as well as test-half 1 at time points 4 and 12 due to a different number of observed categories for the respective test halves and time point combination. Latent means can still be compared across time for the state factors based on the respective other test half. At time point 7, thresholds of both test-halves could not be set invariant across time (due to a divergent number of observed categories). Latent mean differences for the latent state variable at time point 7 should therefore be interpreted with caution.

Supplementary Figure 10 visualizes the latent state means and reliability estimates from the LS model. Reliability was consistently acceptable to good. Only one of the latent means was significantly different from zero, suggesting robust task-level performance and no systematic mean change over time. Supplementary Figure 11 gives the correlations between the latent states for the different time points. Correlations were generally very high, indicating stable individual differences.

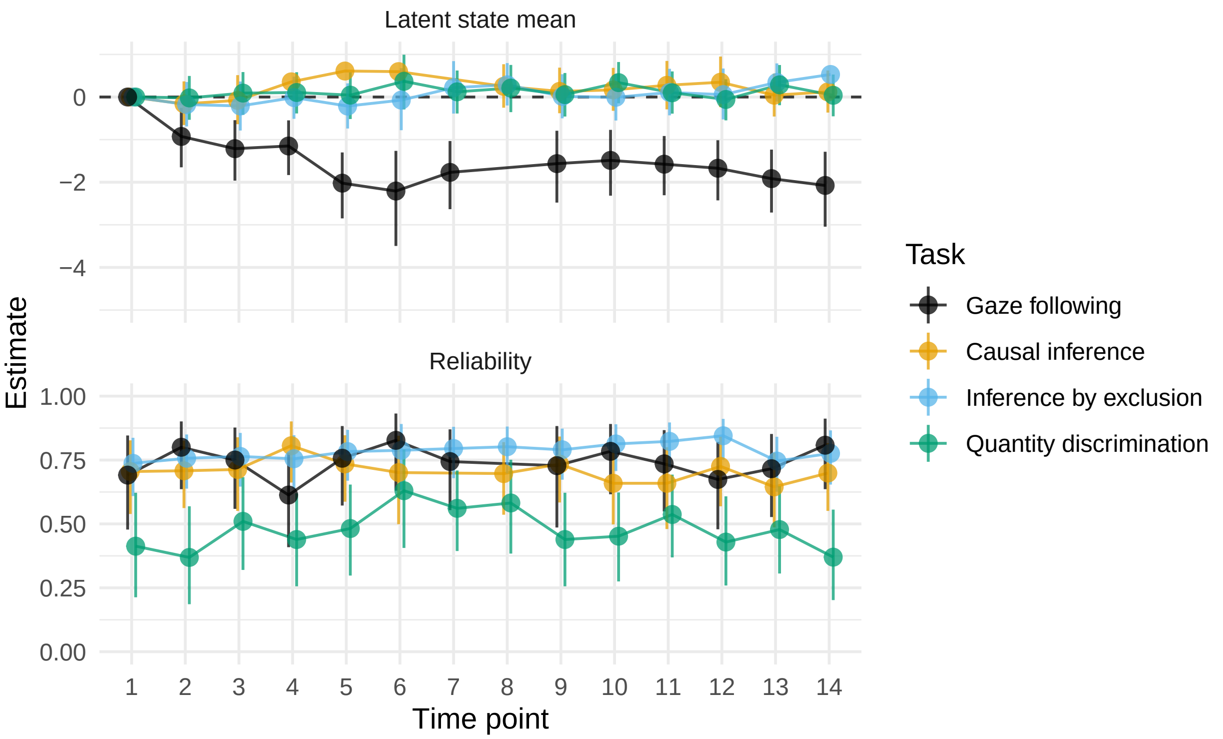

Supplementary Figure 10: Phase 1: latent means and reliability estimates with 95% CI for each time point based on LSM. The sample size varied between time points and can be found in Supplementary Figure 1. Means at time point 1 are set to 0.

In the LST model, the consistency coefficient was estimated to be around .903. This means that around 90% of true inter-individual differences are attributable to stable (trait) differences between individuals, while approximately 10% are due to variance in time point specific deviations from the stable trait. Reliability (across time points) was estimated to be acceptable to good with a mean of .725 (see Supplementary Figure 12).

In sum, all models converge on the conclusion that group- and individual-level performance was highly stable over time. As noted above – and as can be seen in Supplementary Figure 8 – performance on a task level was clearly above chance.

Inference by exclusion Thresholds could not be set equal for test half 2 at time point 6 as well as test half 1 at time points 7 and 14, due to a different number of observed categories for the respective test halves and time-point combination. Latent means can still be compared across time for the respective state factors based on the other test half.

Reliability was good in the LS model and none of the latent means differed from zero (Supplementary Figure 10). Correlations between latent states were generally high across time points (Supplementary Figure 11).

In the LST model, consistency was estimated to be around .859 – around 86% of true inter-individual differences were attributable to stable (trait) differences between individuals. Approximately 14% were due to variance in time-point specific deviations from the stable trait. Reliability was good with an estimate of .815 (see Supplementary Figure 12).

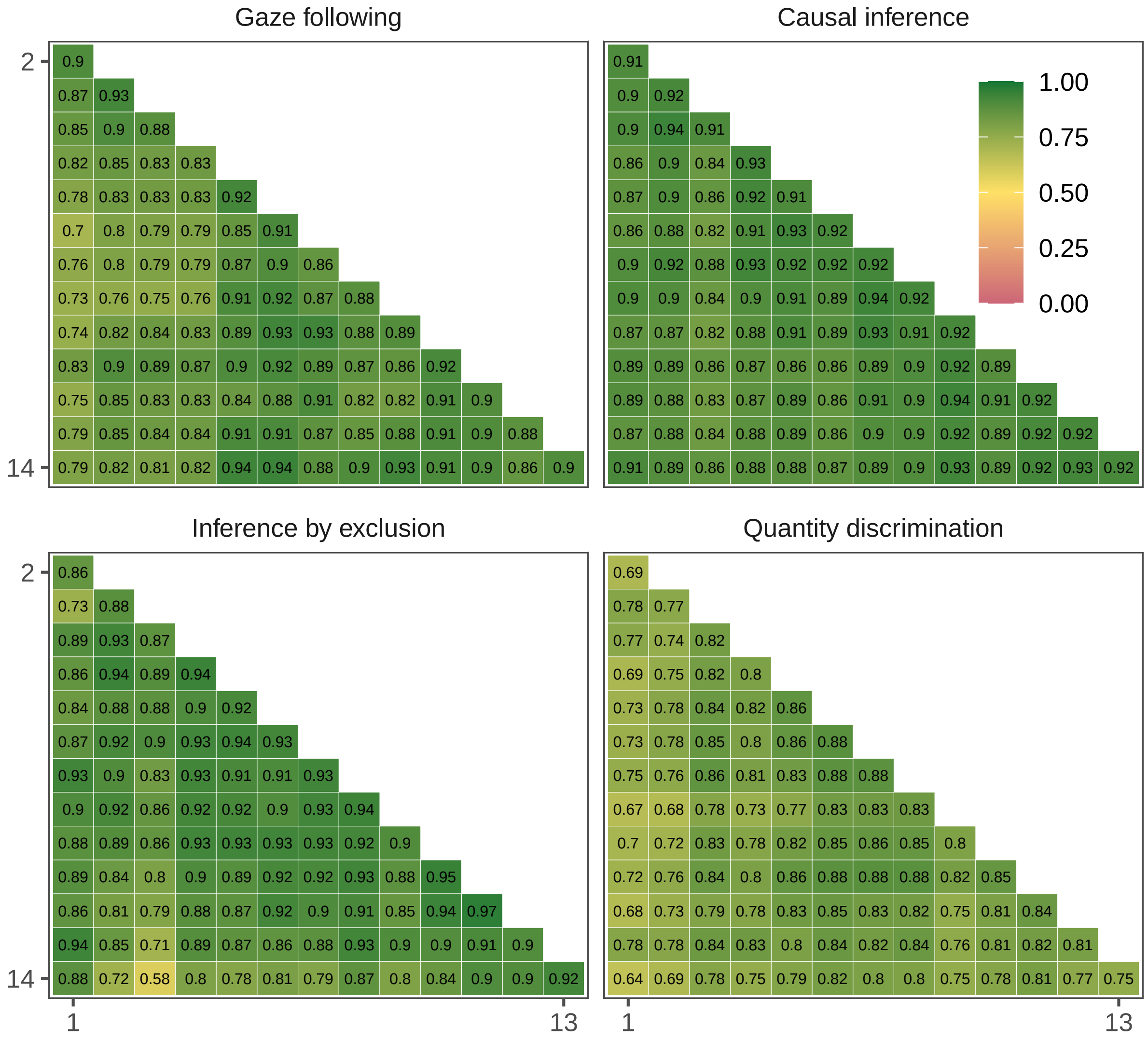

Supplementary Figure 11: Phase 1: correlations between latent state variables based on LSM for the different tasks.

Taken together, we saw a similar pattern as for the direct causal inference task: Performance was very robust on a task level and so were the differences between individuals. Interestingly, from Supplementary Figure 8 we take that task-level performance was at chance. The stable individual differences we found here suggest that variation around this mean was systematic and therefore that some individuals consistently performed above chance. Thus, despite the fact that this task was very difficult for apes, it was suitable to measure individual differences.

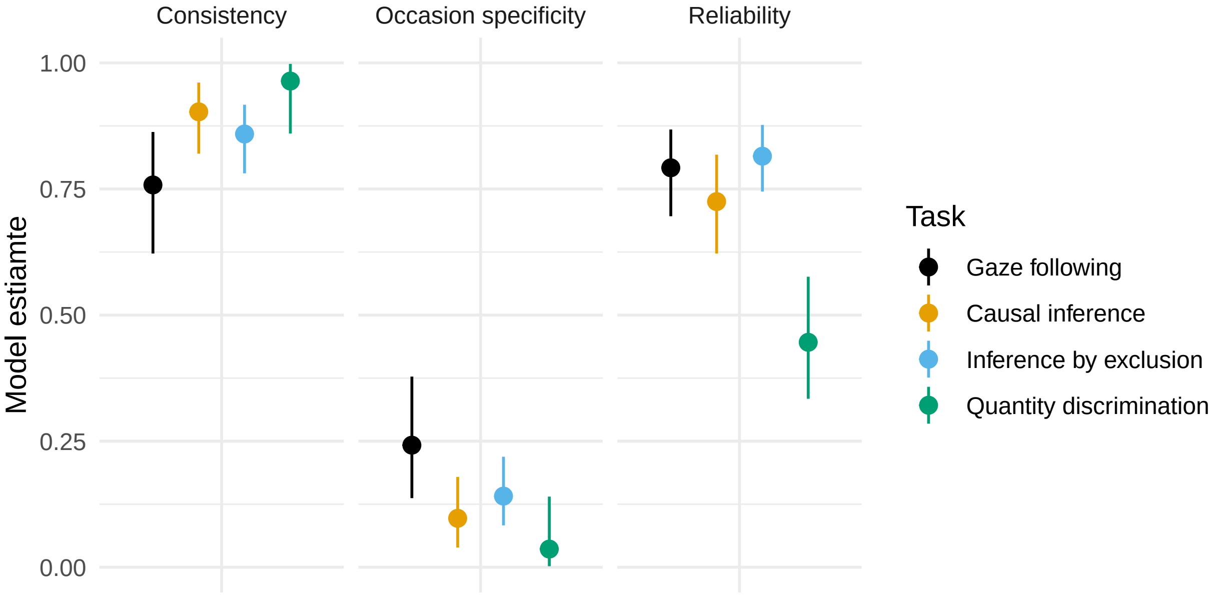

Supplementary Figure 12: Phase 1: Posterior mean for model parameters (with 95% CrI) from LSTM for the four tasks based on data from N = 43 participants.

Gaze following For gaze following, we had only 8 observed trials per measurement occasion. The highest two categories (3 and 4 correctly solved trials) were collapsed into one category due to sparsity. Thresholds could not be set equal for test half 2 at time point 9 as well as test half 1 and test half 2 at time point 8, due to a different number of observed categories for the respective test half and time point combination. Latent means can still be compared across time with the exception of time point 8.

Latent state means estimated in the LS model varied between -0.990 and -2.153. All of the latent state means were significantly lower than zero, suggesting a decrease in gaze following after the second time point (Supplementary Figure 10). Reliability was acceptable to good for all time points. The correlations between latent states for the different time points were generally high, pointing to stable individual differences (Supplementary Figure 11).

In the LST model, consistency was estimated to be around .758, that is around 76% of true inter-individual differences are attributable to stable differences between individuals. Approximately 24% of inter-individual differences were due to variance in time point specific deviations from the stable trait. Reliability was good with an estimate of .792.

In sum, we see a change in gaze following over time (Supplementary Figure 8). This task-level effect, however, did not affect differences between individuals, which were systematic across time points.

Quantity discrimination The lowest three (out of seven possible) categories (0, 1 and 2 correctly solved trials per test half) were collapsed due to sparsity. Thresholds could not be set equal for test half 1 at time point 5, due to a different number of observed categories for the respective test half and time-point combination. Latent means can still be compared across time.

Supplementary Table 3: Model fit indices Phase 2

Task	Model	PPP	Chi 95% CI
Causal inference	LSM	0.222	-73.21 ; 152.76
	LSTM	0.131	-49.19 ; 175.06
Inference by exclusion	LSM	0.168	-88.00 ; 137.28
	LSTM (2 parts)	0.073	-30.08 ; 191.42
Gaze following	LSM	0.470	-107.39 ; 112.12
	LSTM	0.473	-107.46 ; 125.97
Quantity discrimination	LSM	0.266	-77.80 ; 143.97
	LSTM	0.165	-49.70 ; 165.68
Delay of gratification	LSM	0.447	-108.39 ; 117.81
	LSTM	0.207	-65.70 ; 161.37

Note:

LSM = Latent state model

LSTM = Latent state-trait model

PPP = Posterior predictive p-value. Cut-off values (two-tailed): 0.025 and 0.975

Chi 95% CI = 95%CI of difference between predicted and observed chi-square values

Latent state means estimated in the LS model varied very little and all lay between -0.058 and 0.369. None of these state means differed from zero (Supplementary Figure 10). Reliability estimates were substantially lower compared to the other tasks.

The consistency coefficient was estimated to be around .964, that is around 96% of true inter-individual differences was attributable to stable differences between individuals and only approximately 3.6% were due to variance in time-point specific deviations from the stable trait. Again, reliability was rather low with an estimate of .446.

Taken together, quantity judgments were very robust over time on the task level (see also Supplementary Figure 8). The low reliability estimates suggest, however, that the task is less suited to capture individual differences.

Phase 2

For a visual overview of task-level performance in the different tasks see Supplementary Figure 8. The pattern observed in Phase 1 was similar in Phase 2 for causal reasoning and quantity discrimination. The rate of gaze following remained on the low level reached at the end of Phase 1. For inference by exclusion, there was an increase in performance over time, which already began to show itself at the end of Phase 1. Taken together, there was a lot of continuity between Phase 1 and 2. The newly added delay of gratification task produced fairly variable results – with no ceiling or floor effect.

Correlations between time points were similar in Phase 1 and 2 for all tasks (Supplementary Figure 9). Performance in the delay of gratification task proofed to be stable between individuals. Next, we present the results from the SEMs separate for each task. All models showed acceptable fit indices, except for the LST model for inference by exclusion (seeSupplementary Table 3). We therefore adjusted the LST model for this task in a way we describe below. The threshold parameters for each model are shown in Supplementary Table 4.

Direct causal inference As in phase 1, the lowest two categories (0 and 1 correctly solved trials per test half) were collapsed due to sparsity. Thresholds could not be set equal for test half 1 at time point 5, 8, and 11 as well as test half 2 at time point 14 (of phase 2), due to a different number of observed categories for the respective test half and time-point combination. Latent means can still be compared across time.

Supplementary Table 4: Threshold parameters Phase 2

Task	Model	T1	T2	T3	T4	T5	T6
Causal inference	LSM	-3.239	-1.966	-1.337	-0.405	0.764	
	LSTM	-3.026	-1.796	-1.185	-0.274	0.843	
Inference by exclusion	LSM	-1.905	-1.107	0.393	1.229	2.445	
	LSTM	-2.179	-1.358	0.18	1.029	2.249	
Gaze following	LSM	0.393	1.422	2.664			
	LSTM	0.823	1.956	3.279			
Quantity discrimination	LSM	-1.53	-0.947	0.101	1.146		
	LSTM	-1.469	-0.918	0.056	1.015		
Delay of gratification	LSM	-2.099	-1.054	-0.248	0.445	1.335	2.31
	LSTM	-2.554	-1.329	-0.369	0.452	1.508	2.631

Note:

LSM = Latent state model

LSTM = Latent state-trait model

LSTM-AR = LST model with autoregressive component

T1-6 = Threshold parameters for response categories

Supplementary Figure 13 visualizes the latent state means and reliability estimates from the LS model. Reliability was consistently acceptable to good. Except for time point 2, none of the latent means was significantly different from zero, suggesting robust task-level performance and no systematic mean change over time. Supplementary Figure 14 gives the correlations between the latent states for the different time points. Correlations were generally high – indicating stable individual differences – with a trend towards lower correlations for time points further apart.

In the LST model, the consistency coefficient was estimated to be around .912. This means that around 91% of true inter-individual differences are attributable to stable (trait) differences between individuals, while approximately 9% are due to variance in time point specific deviations from the stable trait. Reliability was estimated to be adequate with a value of .726 (see Supplementary Figure 15).

In sum, the results suggest that task-level results were robust and individual-level performance was relatively stable over time. As noted above – and as can be seen in Supplementary Figure 8 – performance on a task level was clearly above chance.

Inference by exclusion The lowest two categories (0 and 1 correctly solved trials per test half) were collapsed due to sparsity. Thresholds could not be set equal for test half 1 at time point 13, due to a different number of observed categories for the respective test half and time-point combination. Latent means can still be compared across time.

The latent state means shown in Supplementary Figure 13 reflect the increase in performance over time that was also noted in Supplementary Figure 8. From time point 6 onward, all latent state means were estimated to be significantly different from zero. Reliability was consistently good. The latent state correlations shown in Supplementary Figure 14 suggest that there was a split in the data in that there were high correlations between latent states among the time points smaller than 8 as well as among time points larger than 8. Looking at Supplementary Figure 8, we can see that the increase in task-level performance started around that time and was largely driven by individuals from the B-chimpanzee group. What this suggests is that some individuals improved in performance and then retained a higher level for the rest of the study. Some of the orangutans changed in the other direction, though to a lesser extend.

With a PPP of 0.002, the LST model for inference by exclusion did not provide a good fit to the data. We suspect the change in performance by some individuals that we discussed above to be responsible for that. That is, the assumption that a single, time invariant trait underlies performance at all time points seemed

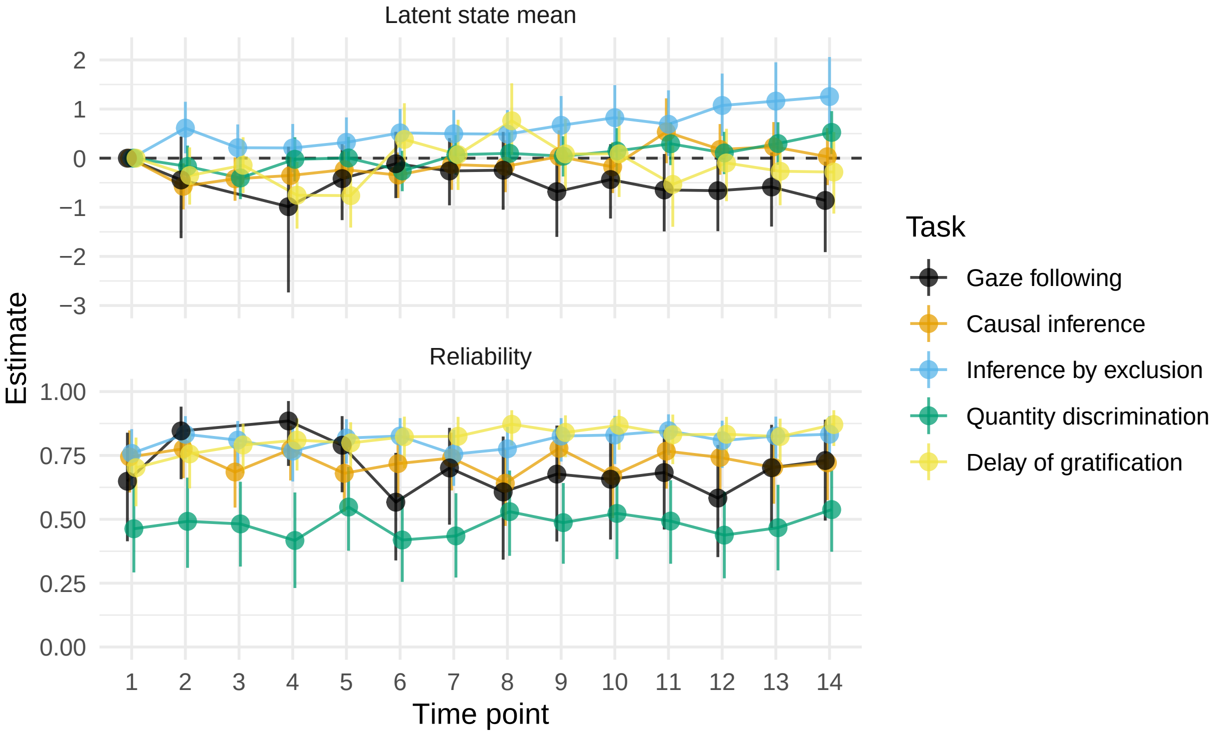

Supplementary Figure 13: Phase 2: latent means and reliability estimates with 95% CI for each time point based on LSM. The sample size varied between time points and can be found in Supplementary Figure 1. Means at time point 1 are set to 0.

to be violated. We therefore fit an adjusted LST model for which we estimated one trait for time points 1 to 8 and a second trait for time points 9 to 14. As a consequence, we get two values for each LST model coefficient (one for each part) as well as a correlation between the two traits.

The adjusted model provided an acceptable fit to the data (see Supplementary Table 3). The consistency coefficients were very similar for the two parts: .912 (Part 1) and .928 (Part 2). That is, for both parts, more than 90% of true inter-individual differences are attributable to stable (trait) differences between individuals, while less than 10% are due to variance in time point specific deviations from the stable trait. The latent trait from the two parts correlated to $r = .815$. The mean of the latent trait variable of part 2 was 0.625 (that is, 0.625 higher than in part 1) and significantly different from zero. Reliability was estimated to be good for both parts: .800 (Part 1) and .830 (Part 2) (see Supplementary Figure 15).

In sum, the models suggest that there was an increase in task-level performance over time which resulted in consistent above-chance performance in later time points (see Supplementary Figure 8). This change was apparently driven by a few individuals who quickly improved and thereby changed their relative ranks. This resulted in a split in the data, with inter-individual differences in each part being quite stable.

Gaze following As in phase 1, the highest two categories (3 and 4 correctly solved trials) were collapsed into one category due to sparsity. Thresholds could not be set equal for test half 2 at time point 1, 4, and 8, test half 1 at time point 9, as well as test half 1 and 2 at time point 3, due to a different number of observed categories for the respective test half and time point combination. Latent means can still be compared across time with the exception of time point 3.

None of the latent state means – except for time point 14 – were significantly different from zero, suggesting a robust average level of gaze following in Phase 2 (Supplementary Figure 13). Reliability varied between acceptable and high, ranging between 0.567 and 0.885. The correlations between latent states for the different time points were generally high, pointing to stable individual differences (Supplementary Figure 14).

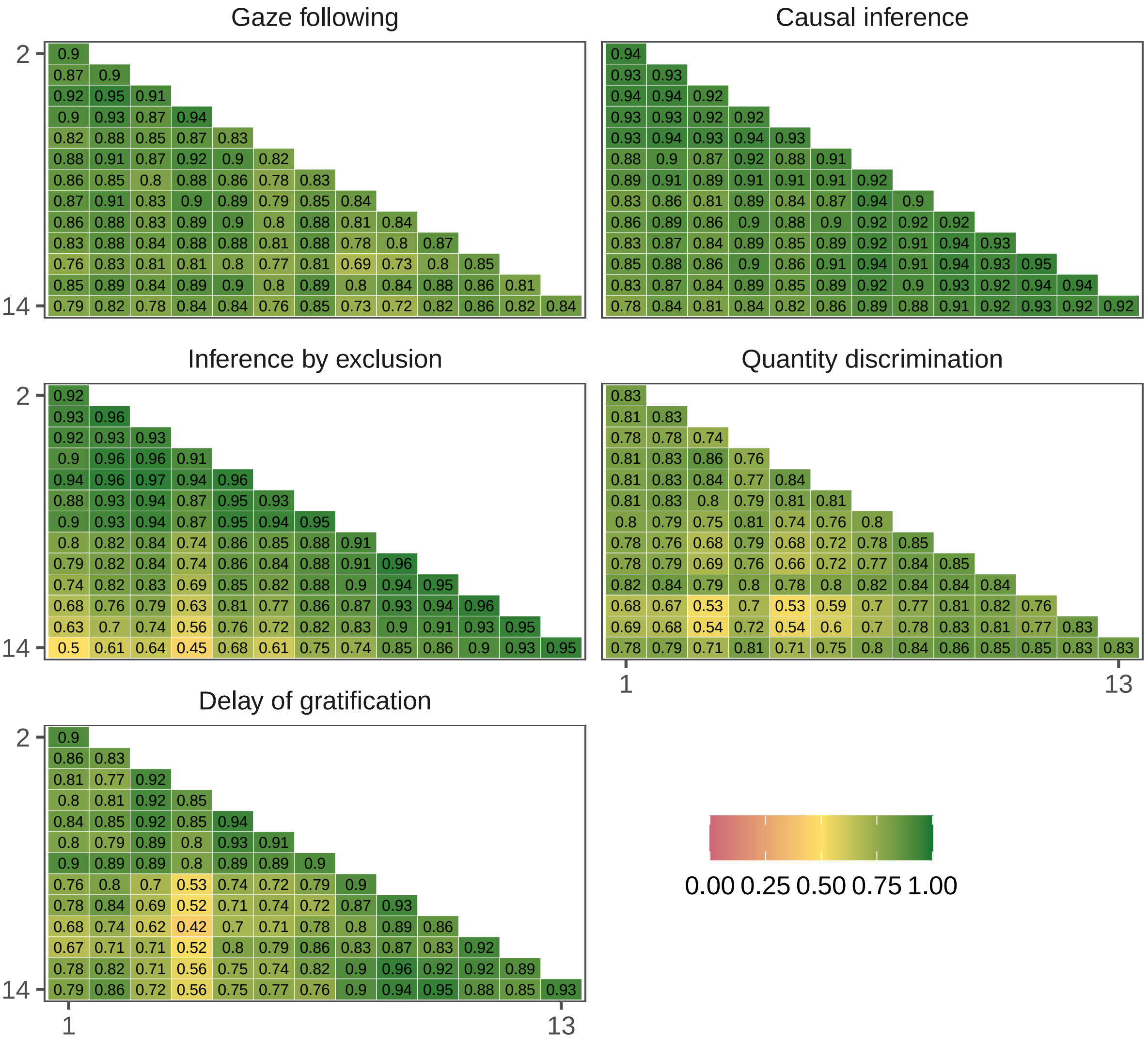

Supplementary Figure 14: Phase 2: correlations between latent state variables based on LSM for the different tasks.

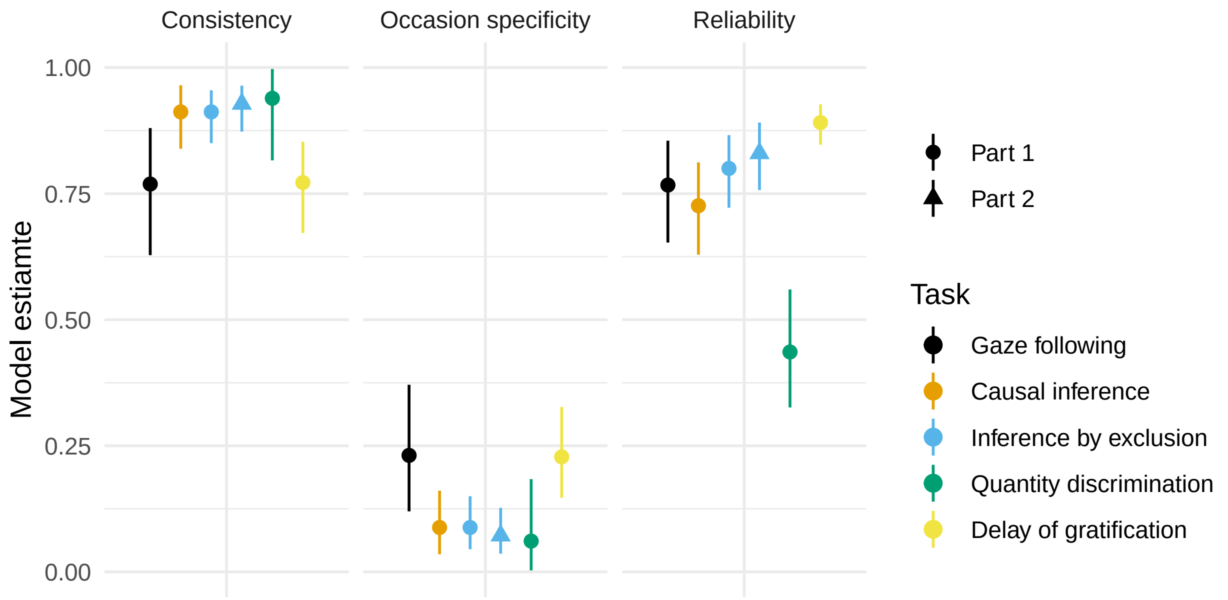

Supplementary Figure 15: Phase 2: posterior means of model parameters (with 95% CI) from LST model for the four tasks based on data from N = 43 participants.

In the LST model, consistency was estimated to be 0.769, that is around 77% of true inter-individual differences are attributable to stable differences between individuals. Approximately 23% of inter-individual differences were due to variance in time point specific deviations from the stable trait. Reliability was good with an estimate of 0.77. In sum, gaze following in Phase 2 was stable over time with systematic and stable differences between individuals (Supplementary Figure 8).

Quantity discrimination As in phase 1, the lowest three (out of seven possible) categories (0, 1 and 2 correctly solved trials per test half) had to be collapsed due to sparsity. Thresholds were set equal across all time points.

Like for gaze following, none of the latent state means differed from zero, except for time point 14. Reliability estimates were substantially lower compared to the other tasks (Supplementary Figure 13). Correlations between time points were generally high, but somewhat lower compared to gaze following or direct causal inference.

In the LST model, the consistency coefficient was estimated to be 0.939, that is around 94% of true inter-individual differences was attributable to stable differences between individuals. Only 6% of inter-individual differences were due to variance in time-point specific deviations from the stable trait. With an estimate of 0.436, reliability was once again lower compared to the other tasks.

Taken together, quantity judgments were robust over time on the task level. Task-level performance was clearly different from chance (Supplementary Figure 8). Performance was also stable on an individual level, with the constraint that the reliability of measurement was lower compared to the other tasks.

Delay of gratification Thresholds could not be set equal for test half 1 at time point 2, 9, 10, 11, and 14 as well as test half 2 at time point 6 and 7 due to a different number of observed categories for the respective test half and time point combination. Latent means can still be compared across time.

Three of the latent state means were significantly different from zero (time point 4, 5 and 8). However, there was no clear temporal pattern in that the latent means steadily de- or increased (Supplementary Figure 13). Reliability was very high at all time points. Latent state correlations across the different time points were

generally high, with time point 4 being a notable exception with lower correlations with time points further away (Supplementary Figure 14).

In the LST model, consistency was estimated to be around 0.772, that is around 77% of true inter-individual differences are attributable to stable differences between individuals. Approximately 23% of inter-individual differences were due to variance in time point specific deviations from the stable trait. Reliability was high with an estimate of 0.891.

In sum, delay of gratification showed some variation in task-level performance. Differences between individuals were relatively stable over time, though slightly less so compared to other tasks (Supplementary Figure 8).

Comparison between phases

For the comparison, we will focus on the four tasks that were included in both phases: direct causal inference, inference by exclusion, gaze following and quantity.

For the direct causal inference task, the raw performance in Supplementary Figure 8 shows no marked difference between the two phases. Similarly, we see no systematic difference between the two phases when looking at the latent state means and threshold parameters estimated by the LS model. This suggests robust task-level performance across the two phases. The LST model estimates for consistency, occasion specificity and reliability were also very similar for the two phases (Supplementary Figure 16), which indicates that individual differences were structured similarly in the two phases.

For inference by exclusion, we saw an increase in performance towards the end of Phase 2 (Supplementary Figure 8), which was also reflected in the latent state means. As noted above, a relatively quick change in performance by some individuals was most likely responsible for this task-level increase. As a consequence, for the LST model, we had to split Phase 2 into two parts. Nevertheless, the LST model estimates for Phase 1 and both parts of Phase 2 were very similar.

The gaze following task showed notable differences between the two phases. The initial decline in gaze following in the beginning of Phase 1 did not repeat itself in Phase 2. Performance in Phase 2 seemed to pick up where it left off at the end of Phase 1 (Supplementary Figure 8). The LS model mean estimates for the different time points follow a similar pattern. Despite this difference in task-level performance, the near-identical LST model estimates for consistency and occasion specificity showed that individual differences were structured in a similar way across the two phases (Supplementary Figure 16).

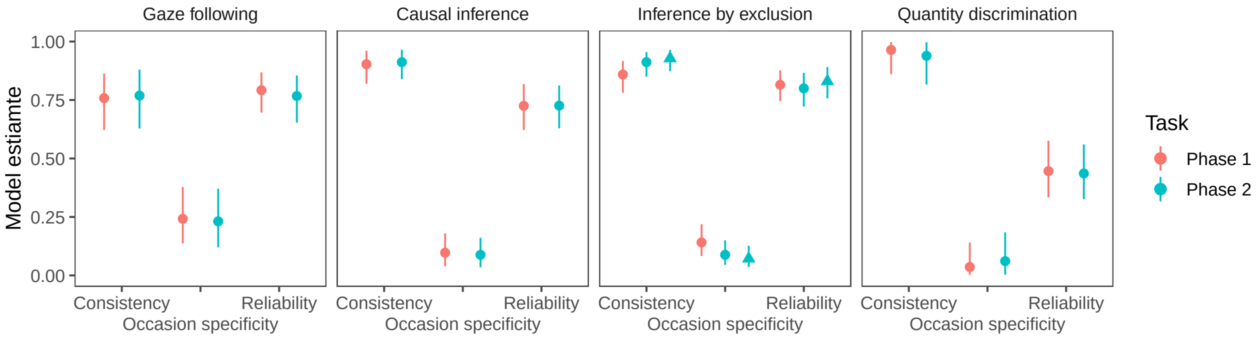

Supplementary Figure 16: LST model estimates for Phase 1 and Phase 2. Points show mean of the posterior distribution with 95% CrI based on data from N = 43 participants. In Phase 2, inference by exclusion has two estimates, one for each part (see text for details).

Raw performance in the quantity task did not show a markedly different pattern in the two phases (Supplementary Figure 8). This was once again also reflected in the latent state means estimated by the LS model and suggests stable task-level performance across the two phases. The three LST model estimates for consistency, occasion specificity and reliability were also very similar for the two phases, which indicates

that individual differences were measured and structured in a similar way in the two phases (Supplementary Figure 16).

Relations between tasks

To analyse relations between different tasks (constructs), we estimated separate LST models, each modeling the relation between two tasks. In these combined models, the sub-models for each task were equivalent to the LST models described above. For ease of model specification, the LST models were estimated as multilevel models. These models are equivalent to the LST models for single tasks under the assumption of strict factorial invariance. Supplementary Figure 17 visualizes the model for two tasks.

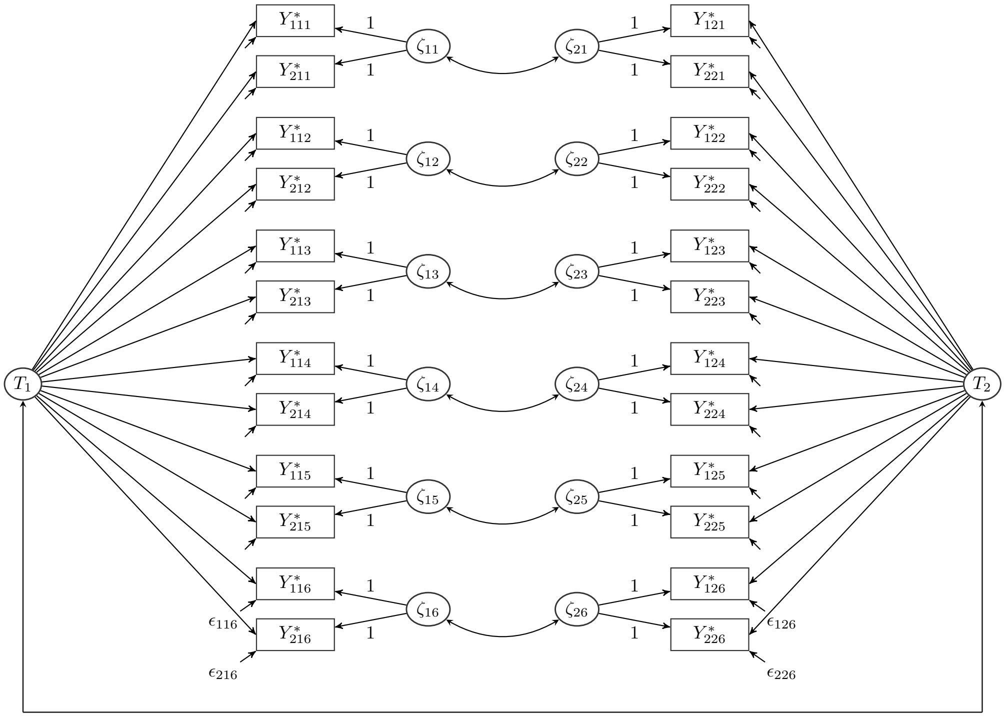

Supplementary Figure 17: Latent State-Trait models for two tasks with correlations between traits and states for two indicators and six measurement time points.

Detailed information on the parameter estimates obtained in LST models for each separate task is provided above. Here we report the results with a focus on the latent correlations only. The parameters of interest were correlations between a) the latent traits, indicating associations between stable cognitive abilities as estimated by the different tasks, and b) correlations between state residual variables belonging to the same measurement time point, as an indicator of time-specific associations between latent abilities across the two tasks, above and beyond stable trait differences.

Simulation studies suggested that LST models in which latent correlations between time-specific state residual variables were estimated to be time-point specific (i.e. covariances and variances of state residual variables can freely vary across time) did not show good estimation performance under the given conditions (sample size, ordinal indicator variables, etc.). Therefore, we chose a model with fixed correlations between state residual variables across time. That is, a model in which we assumed that associations between latent time-specific cognitive abilities across two different tasks within each time point are equal at all time points. We think that this assumption is reasonable in the present context. As a consequence, just one correlation between latent state residual variables is estimated for each model. The corresponding model showed good estimation performance under the given sample sizes in a simulation study.

Supplementary Table 5: Model fit indices for multi-construct models Phase 1

Task1	Task2	PPP	Chi 95% CI
Causal inference	Gaze following	0.371	-17.73 ; 24.37
	Inference by exclusion	0.273	-14.83 ; 28.64
	Quantity discrimination	0.419	-19.26 ; 23.54
Inference by exclusion	Gaze following	0.419	-18.55 ; 24.28
	Quantity discrimination	0.341	-16.10 ; 25.86
Quantity discrimination	Gaze following	0.402	-18.96 ; 23.43

Note:

PPP = Posterior predictive p-value. Cut-off values (two-tailed): 0.025 and 0.975

Chi 95% CI = 95%CI of difference between predicted and observed chi-square values

For details on MCMC estimation see section on estimation above. Because the multi-construct models were considerably more complex (i.e. had more parameters), we increased the minimum number of iterations per Markov chain to 20,000 (with a thinning of 10, that is, 200,000 iterations per chain).

Phase 1

Model fit indices are shown in Supplementary Table 5. Due to a low PPP value, the model for direct causal inference and quantity discrimination was modified such that for each task, test-half specific trait factors were estimated on the between-level. The correlations between the two tasks are therefore also reported as test-half specific trait correlations.

The only correlations for which the 95% CI did not include zero were the state residual correlation between direct causal inference and inference by exclusion (r_{s_c,s_i} = -0.551, 95% CI = [-0.749; -0.299]) and the trait correlation between inference by exclusion and quantity discrimination (r_{t_i,t_q} = 0.436, 95% CI = [0.135; 0.665]).

The negative state correlations between direct causal inference and inference by exclusion may be explained by the way the two tasks were presented. Remember that direct causal inference and inference by exclusion trials used the same setup and were intermixed. A negative correlation suggests that higher (residual) performance in one task was associated with lower performance in the other task. Responding correctly in the two tasks required opposite choice behaviors. That is, in direct causal inference, the ape had to pick the cup the experimenter shook to be correct. In inference, it was the unshaken cup. Such a negative correlation arises when sometimes participants respond in the same way (e.g. pick the shaken cup) across tasks. Note, however, that if this were a stable strategy which individuals would consistently use, we would have seen a negative correlation between the trait estimates. The best explanation is thus that there are short periods of inattentiveness during which (some) participants confused the two tasks.

The trait correlation between inference by exclusion and quantity discrimination was positive, suggesting that individuals with better quantity judgment abilities also have better inferential abilities.

One (out of four) of the test-half specific trait correlations between direct causal inference and quantity discrimination was also different from zero ($r_{t_{c_{h2}},t_{q_{h1}}}$ = 0.466, 95% CI = [0.162; 0.688]). We do not consider this result to be substantial evidence for a substantive association between the trait estimates in the two tasks and therefore do not interpret it any further. Supplementary Figure 18 shows all correlations between the different tasks.

Phase 2

Model fit indices are shown in Supplementary Table 6. Like in Phase 1, due to a low PPP value, the model for direct causal inference and quantity was modified such that for each task, test-half specific trait factors

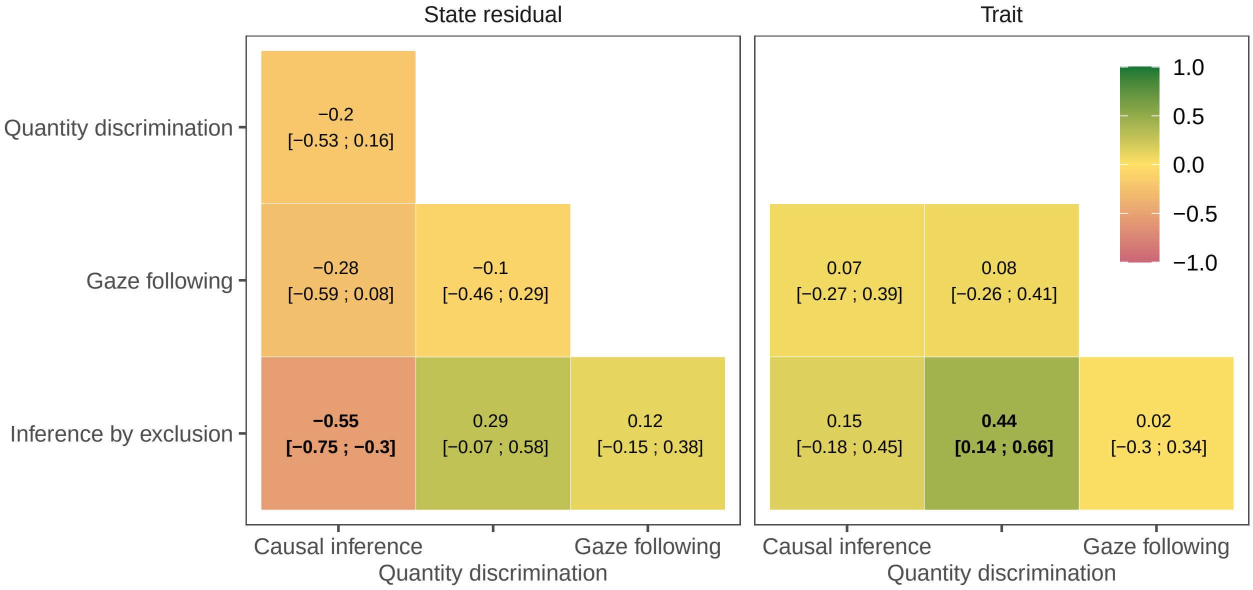

Supplementary Figure 18: Phase 1: correlations between latent traits and latent state residual variables, respectively, of different tasks. Bold correlations are different from zero as judged by the 95% CI. The trait correlations between quantity discrimination and direct causal inference are not displayed, see main text for details. See main text for details.

Supplementary Table 6: Model fit indices for multi-construct models Phase 2

Task1	Task2	Part	PPP	Chi 95% CI
Causal inference	Gaze following		0.588	-23.09 ; 18.68
	Inference by exclusion	Part 1	0.417	-19.74 ; 24.55
		Part 2	0.316	-16.03 ; 26.57
	Quantity discrimination		0.400	-17.66 ; 24.50
	Delay of gratification		0.573	-22.72 ; 18.02
Inference by exclusion	Gaze following	Part 1	0.463	-19.16 ; 22.94
		Part 2	0.302	-16.98 ; 26.43
	Quantity discrimination	Part 1	0.349	-18.18 ; 26.73
		Part 2	0.187	-12.01 ; 31.65
	Delay of gratification	Part 1	0.456	-20.41 ; 22.02
		Part 2	0.298	-15.73 ; 28.00
Quantity discrimination	Gaze following		0.254	-13.93 ; 29.88
	Delay of gratification		0.354	-16.61 ; 26.02
Gaze following	Delay of gratification		0.496	-21.58 ; 21.15

Note:
PPP = Posterior predictive p-value. Cut-off values (two-tailed): 0.025 and 0.975
Chi 95% CI = 95%CI of difference between predicted and observed chi-square values
Part = separate models for the two parts of the data (see main text for details)

were estimated on the between-level. The correlations between the two tasks are therefore also reported as test-half specific trait correlations.

As noted above, we had to fit a 2-part LST model to the data of the inference by exclusion task. We retained this adjustment and for all models involving two tasks, we modeled the correlations between tasks separately for the two parts. Thus, there are two correlations (one for part 1 and one for part 2) for each case involving inference by exclusion.

For the state residuals, the correlation between inference by exclusion and delay of gratification for the first part of the Phase 2 was estimated to be different from zero ($r_{s_{i_1},s_{d_1}}$ = 0.401, 95% CI = [0.08; 0.679]). Given that for the second part of this pairing the correlation was effectively zero ($r_{s_{i_2},s_{d_2}}$ = -0.015, 95% CI = [-0.352; 0.325]), we do not take this to be strong evidence for a systematic relation between the state residuals.

Five of nine task combinations showed evidence for a substantial trait correlation. These included quantity discrimination and delay of gratification (r_{t_d,t_q} = 0.376, 95% CI = [0.066; 0.619]) as well as quantity discrimination and gaze following (r_{t_g,t_q} = 0.353, 95% CI = [0.024; 0.613]). The remaining three included the inference by exclusion task and thus had two coefficients per combination: inference by exclusion and direct causal inference (Part 1: $r_{t_{i_1},t_{q_1}}$ = 0.404, 95% CI = [0.098; 0.639]; Part 2: $r_{t_{i_2},t_{q_2}}$ = 0.449, 95% CI = [0.153; 0.675]) as well as inference by exclusion and delay of gratification (Part 1: $r_{t_{i_1},t_{d_1}}$ = 0.399, 95% CI = [0.098; 0.631]; Part 2: $r_{t_{i_2},t_{d_2}}$ = 0.404, 95% CI = [0.098; 0.643]). For inference by exclusion and quantity discrimination, only the correlation for Part 1 was different from zero, the one for Part 2, however, was also largely positive (Part 1: $r_{t_{i_1},t_{q_1}}$ = 0.526, 95% CI = [0.241; 0.725]; Part 2: $r_{t_{i_2},t_{q_2}}$ = 0.276, 95% CI = [-0.054; 0.554]).

Two (out of four) of the test-half specific trait correlations between direct causal inference and quantity discrimination were also different from zero ($r_{t_{c_{h1}},t_{q_{h2}}}$ = 0.412, 95% CI = [0.106; 0.647]; $r_{t_{c_{h2}},t_{q_{h2}}}$ = 0.342, 95% CI = [0.02; 0.597]). Given that a third correlation was also of similar magnitude ($r_{t_{c_{h2}},t_{q_{h1}}}$ = 0.274, 95% CI = [-0.054; 0.55]), we consider this to be at least suggestive evidence that individuals with higher quantitative abilities were also better at making direct causal inferences. Supplementary Figure 19 shows all correlations between the different tasks.

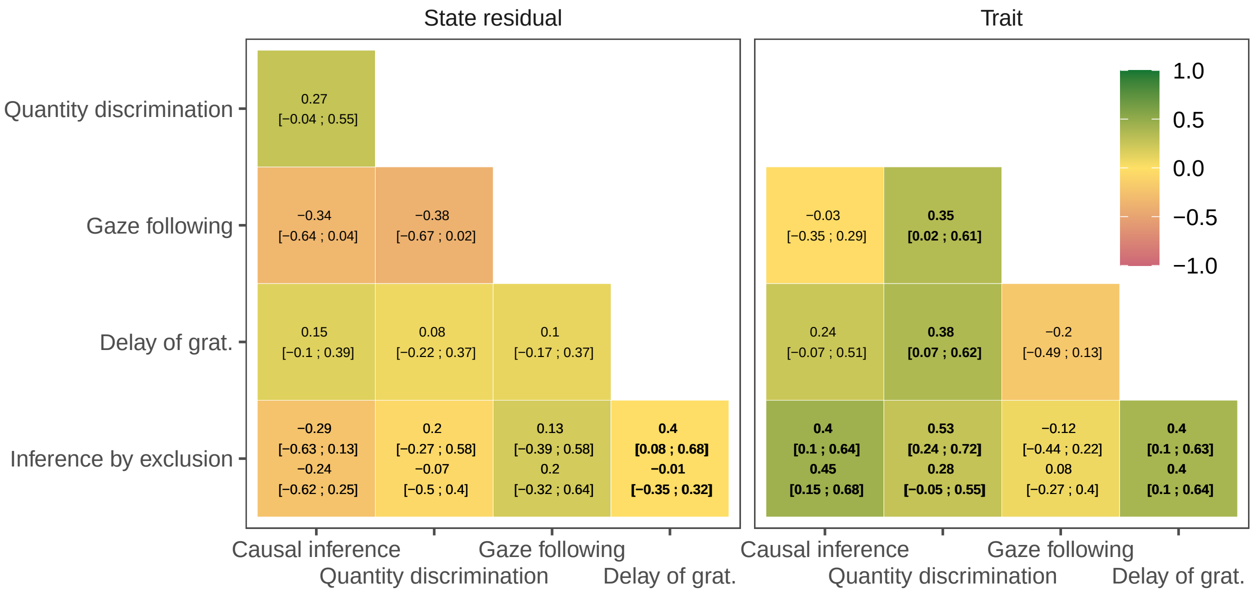

Supplementary Figure 19: Phase 2: correlations between latent traits and latent state residual variables, respectively, of different tasks. Bold correlations are different from zero as judged by the 95% CI.

Comparison between phases

The most notable difference between the two phases was that we saw more substantial trait correlations in Phase 2 compared to Phase 1. This was expected given that we had one additional task in Phase 2 and thus four more combined models. The only significant trait correlation from Phase 1 also turned out to be significant in Phase 2 (quantity discrimination and inference by exclusion). For the tasks that were part of both phases, correlations were numerically similar (Supplementary Figure 20).

These results suggest low to moderate commonalities between the traits – with gaze following being an exception (see Supplementary Figure 20). However, the sample size we studied here is still comparatively small for individual differences research. Larger samples are needed to corroborate these findings.

The significant *negative* state residual correlation between direct causal inference and inference by exclusion found in Phase 1 was not present in Phase 2. This suggests a de-coupling between the two tasks in that apes were less likely to confuse them in Phase 2. This interpretation is also supported by the increase in task-level performance for inference by exclusion and an increase in the trait correlation between the two tasks.

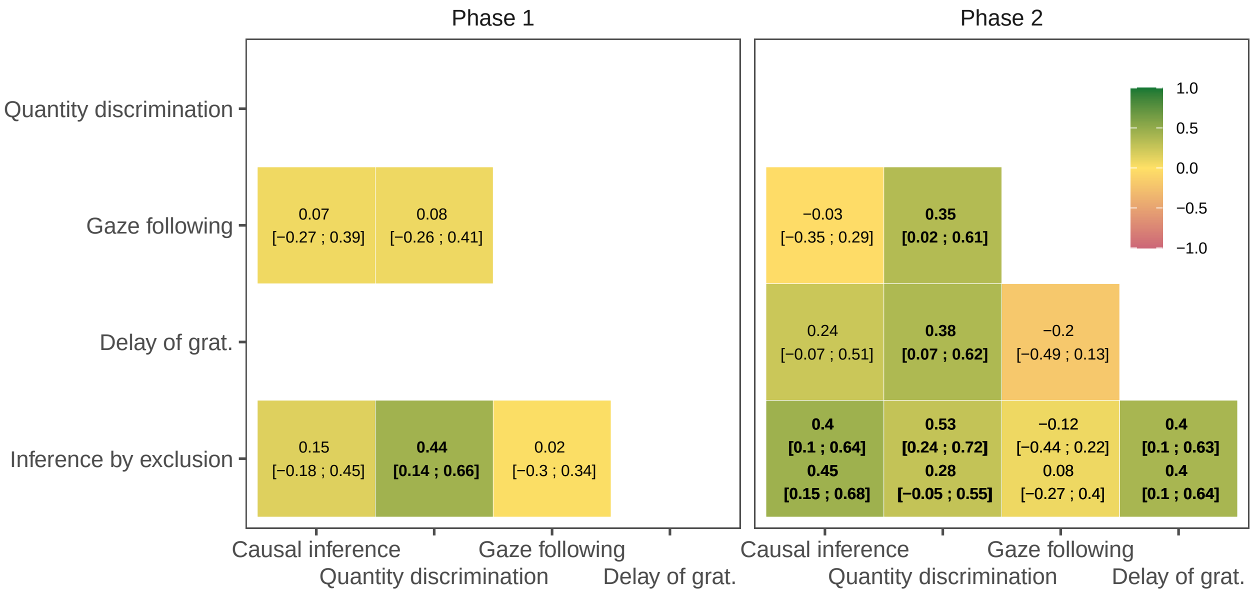

Supplementary Figure 20: Differences between phases in correlations between latent traits and latent state residual variables for the different tasks.

Predictability

The output of the projection predictive inference models is a ranking of the different predictors with respect to how much they improve a model's fit. Predictors ranked first improve the fit the most, while later predictors yield smaller improvements (if any). The selection of "relevant" predictors is based on plotting the loss statistics and looking for a point at which it levels off. As such, the selection is to some extent arbitrary. The ranking, however, is not. When we compare the results from the two phases, we not just look at which subset of predictors is selected, but also at the overall ranking.

Phase 1

Direct causal inference Supplementary Figure 21 visualizes the results. Out of the 13 predictor variables we analysed, we selected only `group` to be relevant in addition to the random intercept term. When inspecting the projected posterior distribution for `group`, we saw substantial differences between the groups: Orangutans

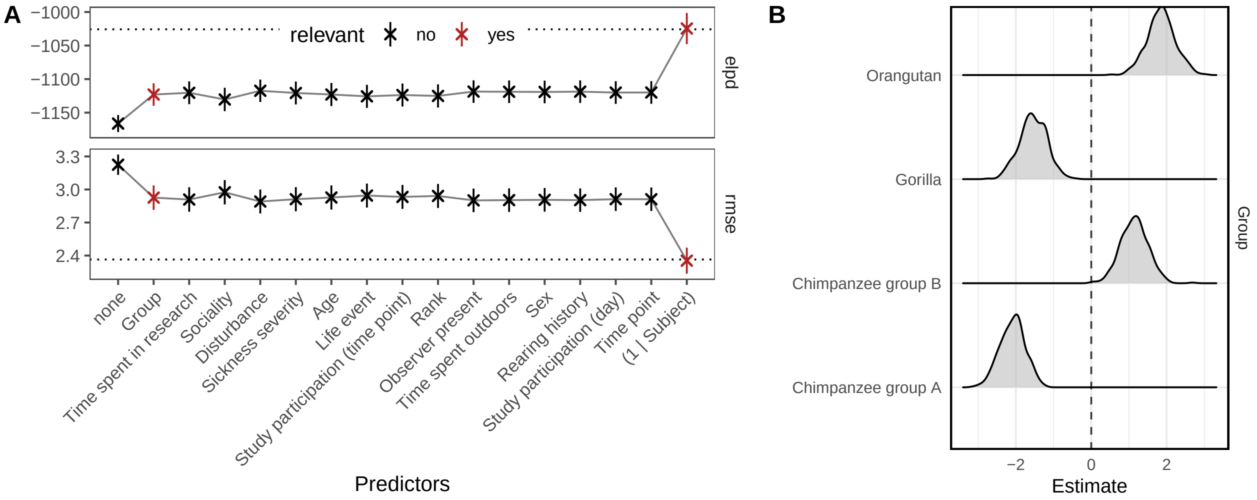

Supplementary Figure 21: Predictor selection for direct causal inference in Phase 1. A) Elpd and RMSE values (with standard error) for predictors based on data from N = 43 participants, ordered by importance (left to right) according to the cross-validated projection prediction model. Note that the random intercept term was forced to be the last one to be included. B) Projections for the selected predictors based on the submodel. Reference level for group are bonobos.

and the B-chimpanzee group performed best, followed by Bonobos (reference level) and finally the Gorillas and the A-chimpanzee group (see Supplementary Figure 21B).

Inference by exclusion Supplementary Figure 22 visualizes the results. For inference by exclusion, we selected `time_in_leipzig`, `group`, and `age` as relevant predictors in addition to the random intercept term. All three predictors capture stable individual characteristics.

Supplementary Figure 22B shows the projected posterior distributions for the predictors and suggests that the longer apes lived in Leipzig, the better their performance was. The differences between groups were such that the two chimpanzee groups together with the Gorillas performed on a higher level compared to the Bonobos (reference level) and Orangutans. With respect to `age`, we found that performance decreased with age.

Gaze Following Supplementary Figure 23 visualizes the results. Gaze following had the most selected predictors of all tasks in Phase 1. In addition to the random intercept term, we selected `group`, `rearing`, `time_outdoors`, `age`, `sociality`, `sex`, `sick_severity`, and `observer`. Half of the predictors were stable individual characteristics, while the other half captured variable characteristics of experience.

Groups differed in that the two chimpanzee groups were most likely to follow gaze followed by the Bonobos (reference level). Gorillas and Orangutans were the least likely to follow the experimenter's gaze. Mother-reared individuals outperformed hand-reared individuals (including those with an unknown rearing history). The more time individuals spent outdoors, the more likely they were to follow gaze. Also, the probability to follow gaze increased with age. Individuals with a lower sociality index had higher rates of gaze following; females outperformed males. The rate of gaze following was lower when observers were present and when higher levels of sickness were reported for individuals. Supplementary Figure 23B visualizes these results.

Quantity discrimination Supplementary Figure 24 visualizes the results. For quantity, we selected three predictors in addition to the random intercept term: `time_in_leipzig`, `rearing`, and `group`. All of these predictors were stable individual characteristics.

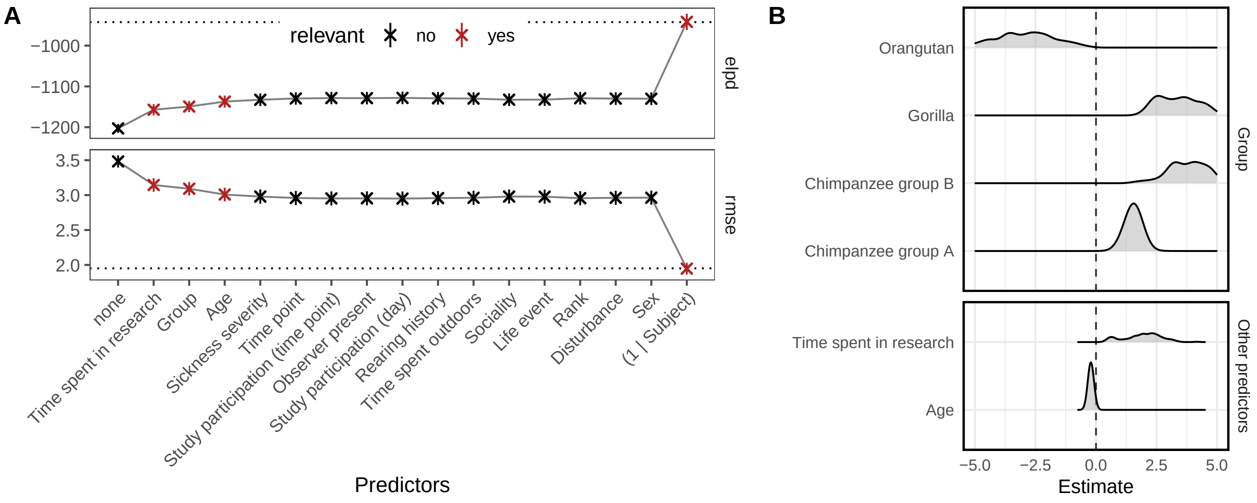

Supplementary Figure 22: Predictor selection for inference by exclusion in Phase 1. A) Elpd and RMSE values (with standard error) for predictors based on data from N = 43 participants, ordered by importance (left to right) according to the cross-validated projection prediction model. Note that the random intercept term was forced to be the last one to be included. B) Projections for the selected predictors based on the submodel.

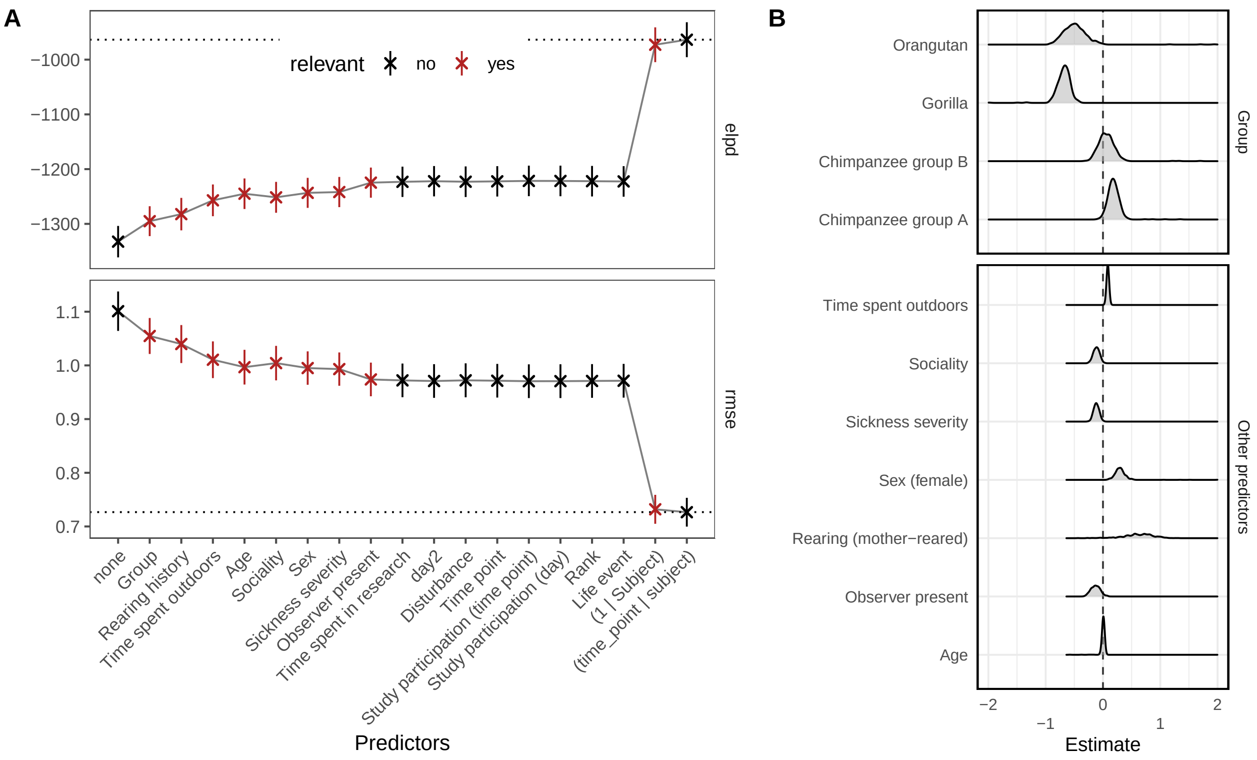

Supplementary Figure 23: Predictor selection for gaze following in Phase 1. A) Elpd and RMSE values (with standard error) for predictors based on data from N = 43 participants, ordered by importance (left to right) according to the cross-validated projection prediction model. Note that the random intercept term was forced to be the last one to be included. B) Projections for the selected predictors based on the submodel model.

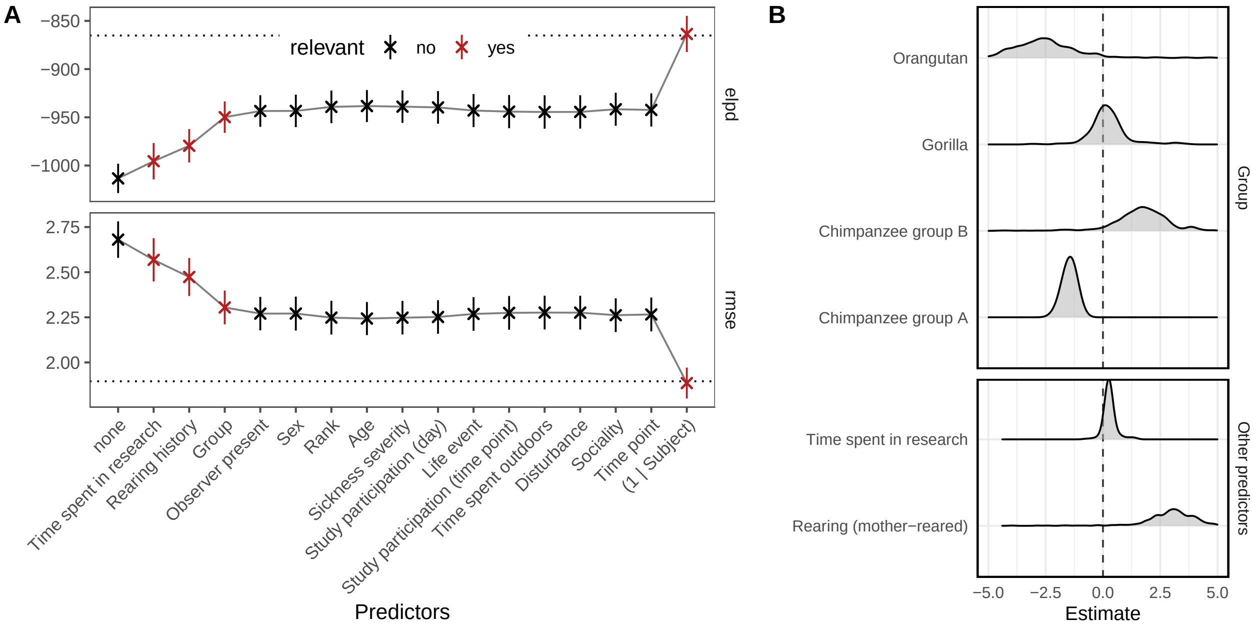

Supplementary Figure 24: Predictor selection for quantity discrimination in Phase 1. A) Elpd and RMSE values (with standard error) for predictors based on data from N = 43 participants, ordered by importance (left to right) according to the cross-validated projection prediction model. Note that the random intercept term was forced to be the last one to be included. B) Projections for the selected predictors based on the submodel model.

The longer individuals had lived in Leipzig, the better they performed in the task. Group differences were such that b-chimpanzees performed best, followed by Bonobos (reference level) and Gorillas. A-Chimpanzees performed slightly worse, but still better than the Orangutans. Once again, mother reared individuals outperformed those who were hand-reared or whose rearing history was unknown. Supplementary Figure 24B visualizes these results.

Summary The most obvious result was that the random intercept term `(1 | subject)` was – by far – the predictor that improved the model fit the most. This suggests that a large portion of the variance is explained by stable individual characteristics that we did not capture in our predictors. Most likely, these are the outcomes of idiosyncratic developmental processes or genetic pre-dispositions, which operate on a much longer time-scale than what we captured in our study.

Second, we saw that most of the relevant predictors came from the group of stable individual characteristics. This aligns well with the SEM results, in which we saw that most of the variance in performance could be traced back to stable trait differences between individuals. Following this reasoning, there was very little *systematic* variation between time points, and thus not much the time-varying predictors could account for. In line with this interpretation, we selected time point specific predictors only for gaze following, the task with the highest occasion specificity estimate according to the LST model.

The predictor selected most often was `group`. Differences between groups were, however, variable. The B-chimpanzee group tended to perform best across tasks, but the ranking of the other groups (including the other chimpanzee group) changed from task to task. This speaks against clear species differences in general cognitive performance. Again, the most likely explanation for group differences is an interaction between species specific dispositions and individual- / task-level developmental processes.

The predictors that were selected more than once influenced performance in a systematic way. Whenever rearing history was selected to be relevant, mother-reared individuals outperformed others. The more time an individual had lived in Leipzig, the better performance was. An exception was `age`, which had a positive

estimate for gaze following but a negative one for inference.

When zooming out, except for `group`, none of the predictors was selected as important for all tasks. As noted above, the relative ranking of the groups varied from task to task.

Phase 2

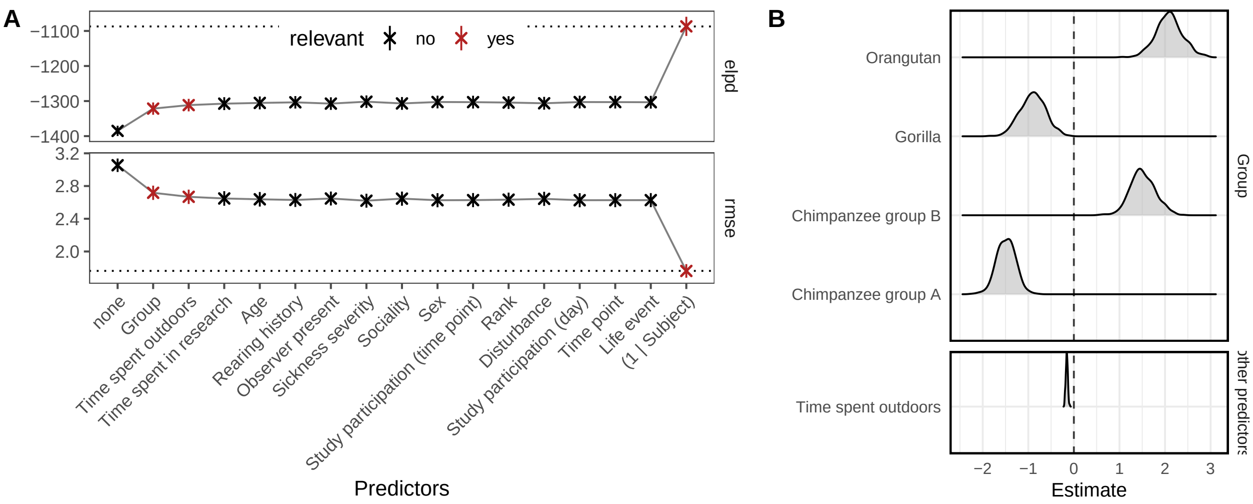

Supplementary Figure 25: Predictor selection for direct causal inference in Phase 2. A) Elpd and RMSE values (with standard error) for predictors based on data from N = 43 participants, ordered by importance (left to right) according to the cross-validated projection prediction model. Note that the random intercept term was forced to be the last one to be included. B) Projections for the selected predictors based on the submodel.

Direct causal inference Supplementary Figure 25 visualizes the results. In addition to the random intercept term, `group` and `time_outdoors` were selected as relevant predictors. When inspecting the projected posterior distribution for `time_outdoors`, we saw that performance was better on time points on which subjects spent less time outdoors. For `group`, we saw substantial differences between the groups: Orangutans and the B-chimpanzee group performed best, followed by Bonobos (reference level) and finally the Gorillas and the A-chimpanzee group (see Supplementary Figure 21B).

Inference by exclusion Supplementary Figure 26 visualizes the results. For inference by exclusion, we selected `time_in_leipzig`, `group`, `age` and `time_point` as relevant predictors in addition to the random intercept term. All predictors)not counting `time_point`) captured stable individual characteristics.

Supplementary Figure 26B shows the projected posterior distributions for the predictors and suggests that the longer apes lived in Leipzig, the better their performance was. For `age` the results suggest better performance for younger individuals. The differences between groups were such that the B-chimpanzee group performed best, followed by the Gorillas. The remaining groups performed on a substantially lower level. The positive effect of `time_point` mirrors the likely learning effects discussed above.

Gaze following Supplementary Figure 27 visualizes the results. In addition to the random intercept term, we selected `group`,`sex`, `observer`, and `age`. All of these predictors were stable individual characteristics, with the exception of `observer`.

Groups differed in that individuals from the A-chimpanzee group were most likely to follow gaze, followed by the B-chimpanzee group and the bonbons (reference level). Gorillas and the Orangutans were less likely to follow gaze. Females outperformed males. The rate of gaze following was higher when no observer was

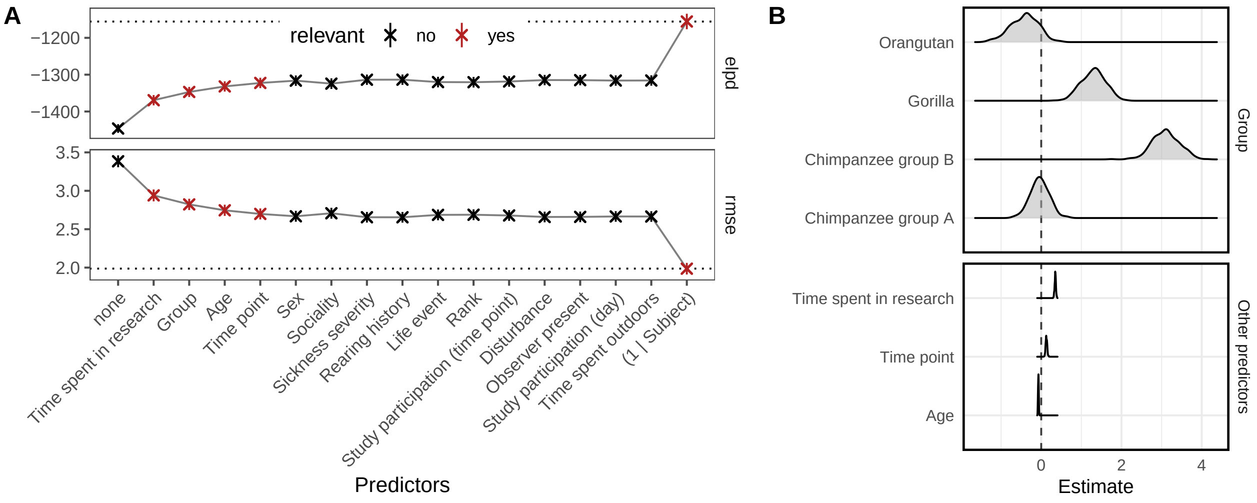

Supplementary Figure 26: Predictor selection for inference by exclusion in Phase 2. A) Elpd and RMSE values (with standard error) for predictors based on data from N = 43 participants, ordered by importance (left to right) according to the cross-validated projection prediction model. Note that the random intercept term was forced to be the last one to be included. B) Projections for the selected predictors based on the submodel.

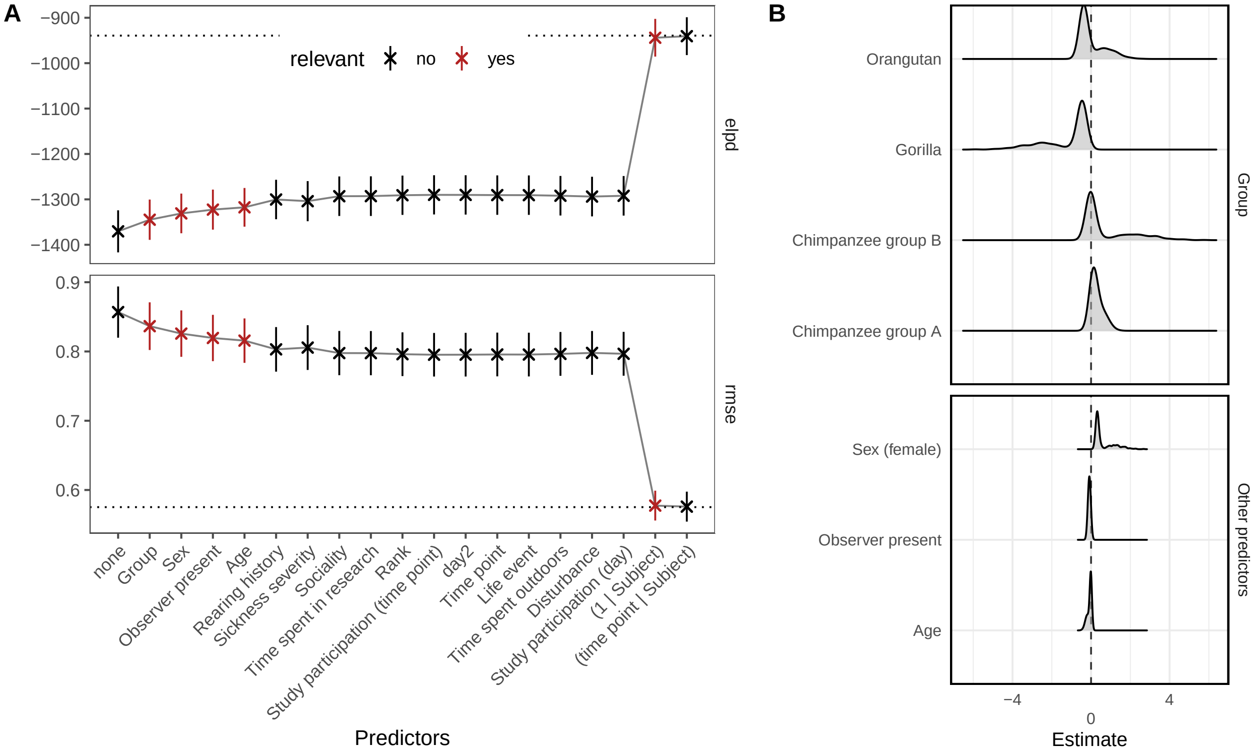

Supplementary Figure 27: Predictor selection for gaze following in Phase 2. A) Elpd and RMSE values (with standard error) for predictors based on data from N = 43 participants, ordered by importance (left to right) according to the cross-validated projection prediction model. Note that the random intercept term was forced to be the last one to be included. B) Projections for the selected predictors based on the submodel model.

present. Finally, the rate of gaze following further decreased with age. Supplementary Figure 27B visualizes these results. Please not the unusual shape of the posterior distribution for some of the predictors. While we are confident about the selection of the selection process, we would caution against an overly strong interpretation of the absolute values of the projected estimates.

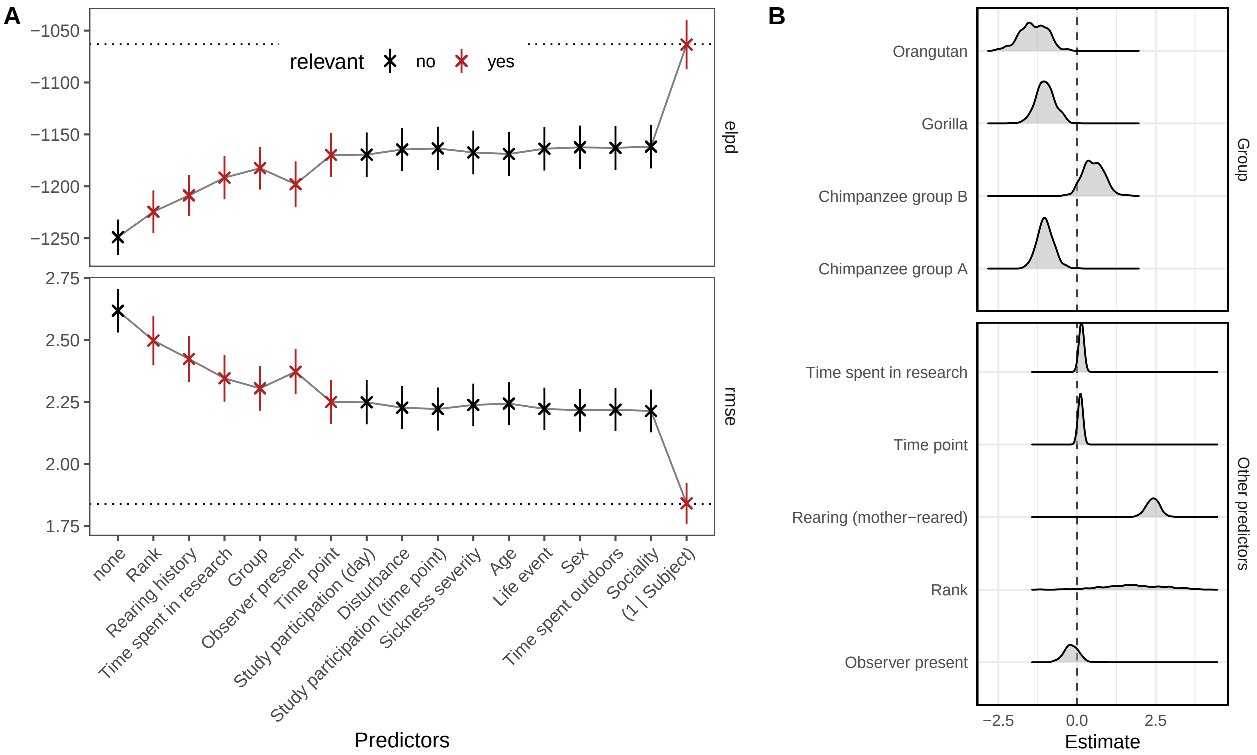

Supplementary Figure 28: Predictor selection for quantity discrimination in Phase 2. A) Elpd and RMSE values (with standard error) for predictors based on data from N = 43 participants, ordered by importance (left to right) according to the cross-validated projection prediction model. Note that the random intercept term was forced to be the last one to be included. B) Projections for the selected predictors based on the submodel model.

Quantity discrimination Supplementary Figure 28 visualizes the results. In addition to the random intercept term, we selected `rel_rank`, `rearing`, `time_in_leipzig`, `group`, `observer` and `time_point`. Two of the five predictors (excluding `time_point`) captured more variable aspects of experience.

Supplementary Figure 26B shows the projected posterior distributions for the predictors. Higher ranking individuals performed better in the task. Mother-reared individuals performed better compared to hand-reared individuals. The more time the individual had spent in Leipzig, the better the performance. With respect to `group`, we found that the B-chimpanzee group performed above the Bonobos (reference level) which in turn were better compared to the other groups. Performance was better, when there was no observer. Finally, individuals who had already participated in another study on the same day showed better performance. The effect of `time_point` suggested a slight increase in performance over time.

Delay of gratification Supplementary Figure 29 visualizes the results. In addition to the random intercept term, we selected `time_in_leipzig`, `observer`, and `sex`. Notably, delay of gratification was the only task for which `group` was not selected as a predictor.

Individuals who have spent more time in Leipzig were more likely to delay gratification. Waiting for the bigger reward was also more likely when there was no observer present. Males also had higher rates of waiting. Supplementary Figure 29B visualizes these results.

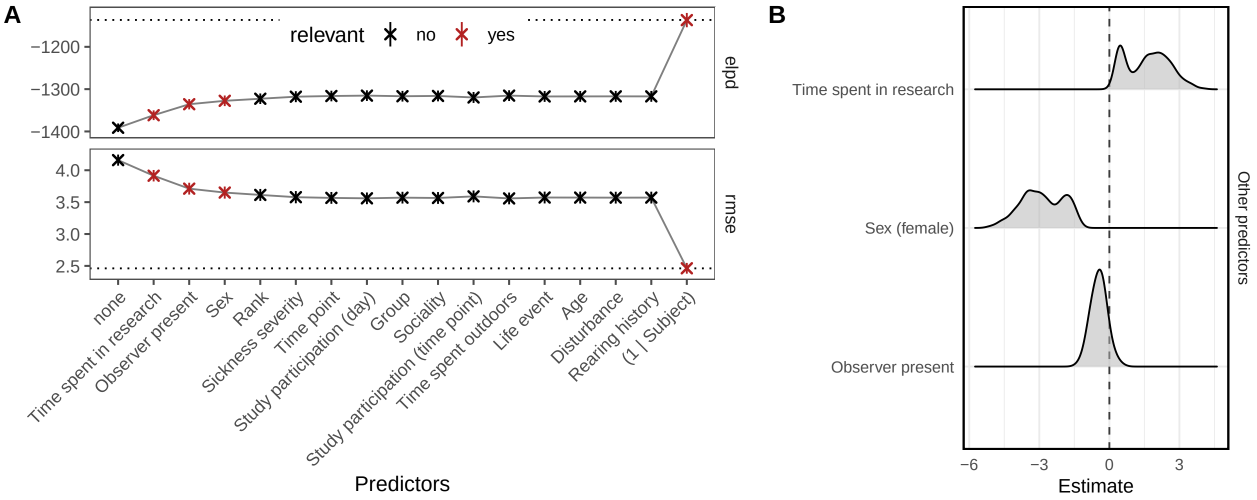

Supplementary Figure 29: Predictor selection for delay of gratification in Phase 2. A) Elpd and RMSE values (with standard error) for predictors based on data from N = 43 participants, ordered by importance (left to right) according to the cross-validated projection prediction model. Note that the random intercept term was forced to be the last one to be included. B) Projections for the selected predictors based on the submodel model.

Summary Once again, the random intercept term `(1 | subject)` was the predictor that improved the model fit the most. Predictors capturing stable individual characteristics were selected most often, which aligns well with the SEM results, in which we saw that most of the variance in performance could be traced back to stable trait differences between individuals. The tasks with the highest occasion-specific variance (gaze following and delay of gratification, see Supplementary Figure 15) were also those for which the most time point specific predictors were selected. However, the quantity discrimination task did not fit this pattern; even though the LST model suggested that only a very small portion of the variance in performance was occasion specific, three time point specific variables were selected to be relevant.

The predictor selected most often was `group`. The differences between groups were, however, variable with no obvious pattern. The two chimpanzee groups varied largely independent from each other. Thus, it makes more sense to interpret this as differences between groups rather than species.

The way in which the predictors that were selected more than once influenced performance was largely coherent. The presence of observers always had a negative effect on performance. The more time an individual had lived in Leipzig, the better performance was. Higher-ranking individuals outperformed lower-ranking ones. Finally, younger individuals outperformed older ones. An Exception was `sex`: females were more likely to follow gaze than males, but males were more likely to wait for the larger reward in the delay of gratification task.

Taken together, there was no single predictor that was selected as important for all tasks. The most important predictor across tasks was `group`, but the relative ranking of groups was not consistent across tasks.

Comparison between phases

Supplementary Figure 30) shows the predictor ranking and selection across tasks and phases. For the following comparison between the two phases, we focus on the tasks that were part of both. The picture that emerges is one of consistency with occasional variability.

Inference by exclusion was the most consistent of all tasks, that is, the same predictors were selected to be relevant in both phases (`group`,`time_in_leipzig`, `age`). Furthermore, theses predictors were ranked in the same order.

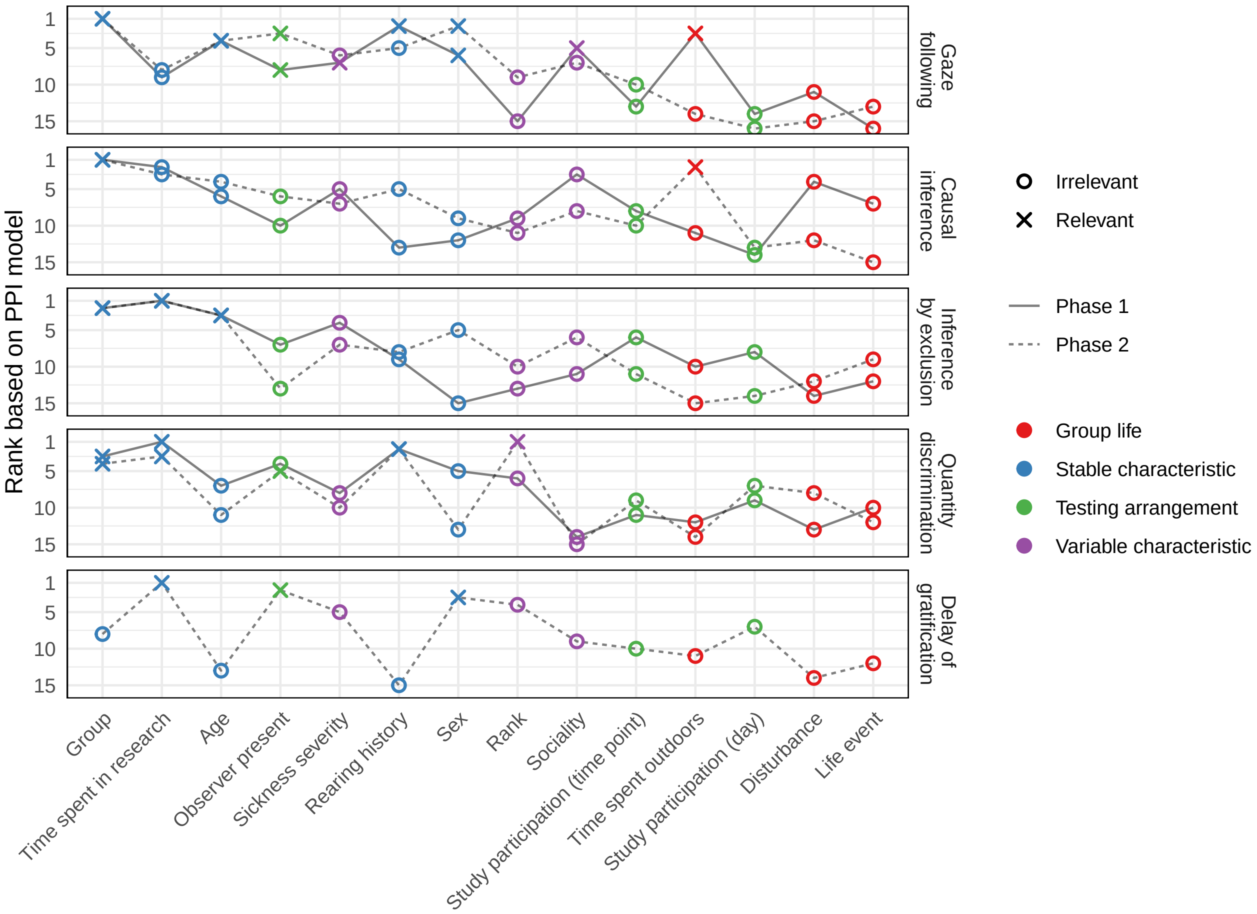

Supplementary Figure 30: Predictor ranking and selection based on PPI models for Phase 1 and Phase 2. Different linetypes show the different phases, crosses mark predictors that were selected to be relevant based on the PPI models. Color shows the broader category each predictor belongs to. The x-axis is sorted by the average rank across tasks and phases.

For direct causal inference, the highest ranked predictor was the same in both phases (`group`). However, in Phase 2, `time_outdoors` was also selected to be relevant even though it had received a fairly low rank in Phase 1.

For quantity discrimination, all predictors that were selected in Phase 1 were also selected in Phase 2 (`time_in_leipzig`, `rearing`, `group`, similar ranking in both Phases), however, Phase 2 two additional – time point specific – predictors:`rank` and ‘`observer`.

The gaze following task was the only one for which some of the predictors selected in Phase 1 were not selected in Phase 2 (`rearing`, `time_outdoors`, `sociality`, `sick_severity`). However, except for `time_outdoors`, despite not being selected, the ranking of the predictors was similar. The divergence for `time_outdoors` can largely be explained by the initial habituation we saw in gaze following at the beginning of Phase 1 (Supplementary Figure 8) which coincided with a seasonal decline in time spent outdoors (Supplementary Figure 5). At the beginning of Phase 2, gaze following rates remained low while time spent outdoors was high again. Thus, the result of Phase 1 is likely to be a mere artifact. Overlapping predictors between phases were `group`, `age`, `observer`, and `sex`. The case of `observer` is the only one in which a variable predictor was selected in both phases.

The consistency between phases was that for most tasks the predictors selected as relevant in Phase 1 were again selected in Phase 2. This constitutes solid evidence that there is a systematic and consistent relation between performance and these predictors. All predictors that were selected twice capture stable individual differences (`group`, `age`, `time_in_leipzig`, `rearing`), echoing once again the results of the LST models. Variability showed itself in the time point specific variables. There was no overlap in time point specific predictors between Phase 1 and 2. This suggests that cognitive performance *is* influenced by temporal variation in group life, testing arrangements and variable characteristics, the way this happens, however, is either less consistent or less pronounced (or both).

The stable individual characteristics that turned out to be systematically related to performance varied across tasks. That is, except for `group`, none of the predictors was relevant for all tasks. The ranking of the different groups varied across tasks, with no clear pattern. That is, none of the groups consistently performed better or worse compared to the others across tasks. There seems to be no one-size-fits-all predictor of cognitive abilities in great apes.

Summary

When reflecting on the results presented here, we can draw the following tentative conclusions: First, the majority of tasks produced robust task-level results and showed relatively high re-test correlations. Exceptions were the switching task – which produced no interpretable individual differences – and the quantity discrimination task, which produced robust task-level results but less reliable individual differences. Thus, assessing the psychometric properties of tasks is an important prerequisite to contextualize and interpret the results of individual differences studies in great apes.

We saw that individual differences in performance were best explained by stable differences in cognitive abilities between individuals. This does not mean that these abilities are time-invariant. For the inference by exclusion task, we saw that some individuals quickly improved, leading to a re-ordering of individual difference. It means that change is systematic and potentially long-lasting, reflecting a change in the underlying cognitive abilities.

Performance was best predicted by variables that captured stable individual characteristics such as group membership, age or experience with research. This aligns well with the stability of individual differences mentioned above. However, the single most important predictor of performance was a term that simply reflected the identity of the individual. This suggests that the – fairly comprehensive – set of predictors that we studied here do not fully explain cognitive performance in great apes. Most likely, idiosyncratic developmental processes in combination with genetic pre-dispositions – which operate on a much longer time-scale – are the source of these unexplained individual differences.

Supplementary Note

Analysis and results

Latent state-trait models with autoregressive effects (LST-AR)

LST-AR models extend LST models by accounting for potential carry-over effects between temporally adjacent observations. The autoregressive effects thereby capture accumulated situational effects, that is, effects of previously experienced situations on subsequent trait levels (Eid et al., 2017). For instance, a person that experiences a great success at work at time t may not only show greater job satisfaction as compared to their habitual trait level at that point in time, but an increase in job satisfaction that lasts across a prolonged time period (i.e., change in the habitual trait level at time t' with $t' > t$ due to the positive experience at time t). LST-AR models allow us to quantify the temporal predictability of performance based on occasion-specific variance in the previous time points. This is captured in the (autoregressive) predictability coefficient and quantifies how much of the variation in performance can be explained by the variation in the occasion-specific variables at the previous time point (note that the names of the coefficients used here slightly differ from those used in Eid et al. (2017)).

Model description The following model is a restrictive variant of the model described in Eid et al. (2017). The model is depicted for six measurement time points in Supplementary Figure 31.

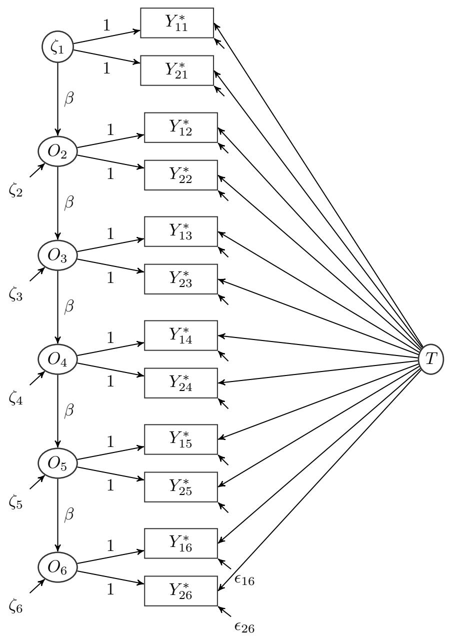

Supplementary Figure 31: Latent State-Trait model with autoregressive effects for two indicators and six measurement time points. All factor loadings of the latent trait factor T are fixed to 1 (not displayed in the figure)

Measurement equation for parcel i at time point t:

$$Y^*_{it} = T + O_t + \epsilon_{it} \tag{8}$$

where T is the latent trait variable, the occasion-specific variables O_t capture time-specific deviations of the respective true score from the latent trait variable T, and ϵ_{it} is a measurement error variable, with

$\epsilon_{it} \sim N(0,1)\ \forall i,t$. O_t is assumed to follow an autoregressive process of order 1 across time (within subjects), that is:

$$O_t = \zeta_t \qquad t = 1$$
$$O_t = \beta O_{(t-1)} + \zeta_t \qquad t > 1$$

where the latent state residual variables ζ_t capture true time-specific inter-individual differences that cannot be explained based on the true score of previous measurement time points. We make the same assumptions about factorial invariance as in the LST model.

The following variance coefficients can be computed. Note that the names of the coefficients used here slightly differ from those used in Eid et al. (2017).

Consistency Proportion of true variance (i.e., measurement-error free variance) that is due to true inter-individual stable trait differences.

$$Con(Y^*_{it}) = \frac{Var(T)}{Var(T) + \beta^2 Var(O_{(t-1)}) + Var(\zeta_t)} \tag{9}$$

Occasion specificity Proportion of true variance (i.e., measurement-error free variance) that is due to true inter-individual differences in the state residual variables, that is occasion-specific variation that is not explained by the autoregressive process or the trait variable T.

$$OS(Y^*_{it}) = \frac{Var(\zeta_t)}{Var(T) + \beta^2 Var(O_{(t-1)}) + Var(\zeta_t)} \tag{10}$$

As the proportion of variance explained by the autoregressive process stabilizes over time, all coefficients have converged to a relatively stable value at $t = 14$, indicating the long-term proportions of variance that are to be expected.

Autoregressive predictability Proportion of true variance that is explained by carry-over effects from previous measurement time points:

$$Pred(Y^*_{it}) = \frac{\beta^2 Var(O_{(t-1)})}{Var(T) + \beta^2 Var(O_{(t-1)}) + Var(\zeta_t)} \tag{11}$$

Results All LST-AR models in Phase 1 and Phase 2 fit the data reasonably well (see Supplementary Table 7). The model estimates for consistency, occasion specificity and reliability were largely similar to the LST model models (see Supplementary Figure 32). There were some pronounced differences: most notably, the models for the inference by exclusion and quantity discrimination tasks had very high predictability estimates in Phase 2. However, these results should be interpreted with caution. First, the credible intervals for the parameter estimates for consistency and predictability were very wide – covering the whole range of possible values between 0 and 1 – suggesting that the model was in fact unable to precisely quantify the two components.

More importantly, however, the β coefficients that quantify the auto-regressive effect suggest that the models were in fact unidentifiable (see also Lüdtke, Robitzsch, & Wagner, 2018). The upper bound for β is 1 (i.e. 100% of true score variance of t_i is predicted by the residual state variance of t_{i-1}) and the 95% CIs for the parameter estimates for the models in question included values that were larger than that (Supplementary Figure 33). This was also the case for the direct causal inference model of Phase 2. Because of this issue, and because the unproblematic models did not yield different interpretations from the LST models, we did not use the LST-AR models to interpret the results.

Supplementary Table 7: Model fit indices for LST-AR models

Phase	Task	PPP	Chi 95% CI
Phase 1	Causal inference	0.262	-80.04 ; 156.56
	Inference by exclusion	0.485	-114.89 ; 126.70
	Gaze following	0.197	-65.37 ; 165.29
	Quantity discrimination	0.520	-116.23 ; 108.54
Phase 2	Causal inference	0.251	-72.14 ; 149.75
	Inference by exclusion	0.457	-106.12 ; 121.36
	Gaze following	0.094	-39.28 ; 189.43
	Quantity discrimination	0.258	-75.81 ; 145.93
	Delay of Gratification	0.333	-85.82 ; 141.26

Note:
PPP = Posterior predictive p-value. Cut-off values (two-tailed): 0.025 and 0.975
Chi 95% CI = 95%CI of difference between predicted and observed chi-square values

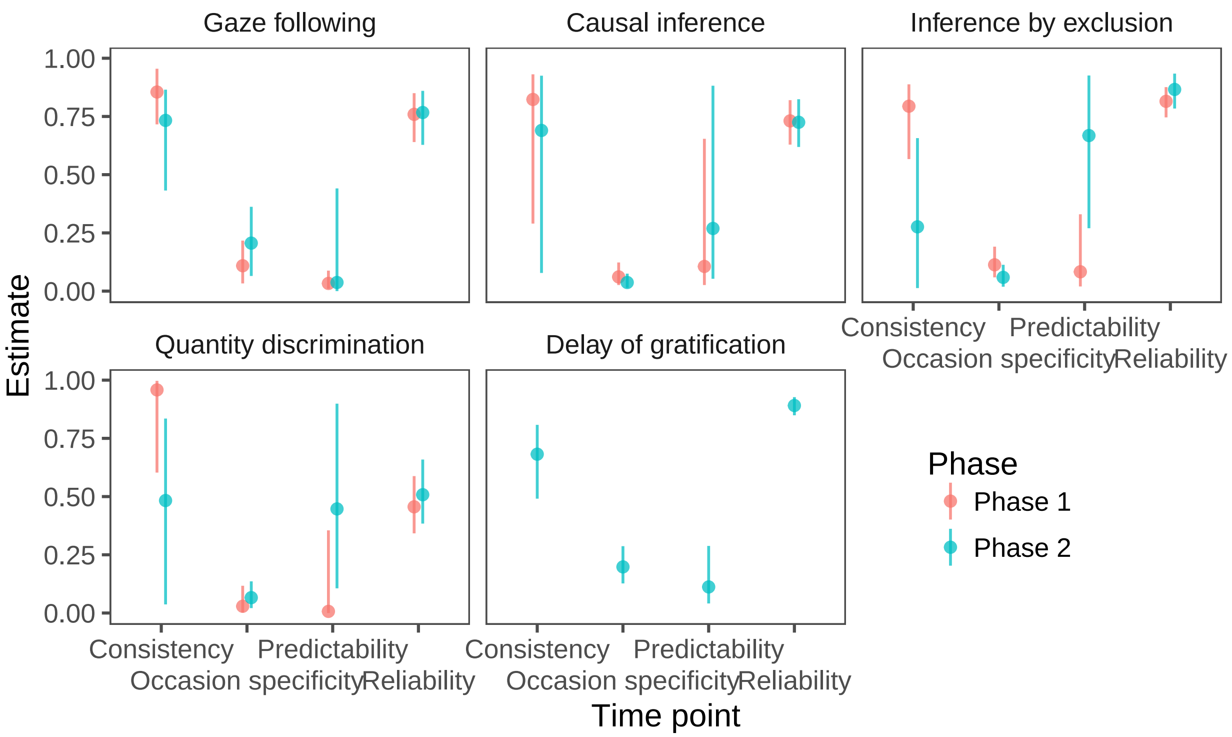

Supplementary Figure 32: Posterior means of LSTM-AR estimates for time point 14 for Phase 1 and Phase 2 with 95% CrI based on data from N = 43 participants.

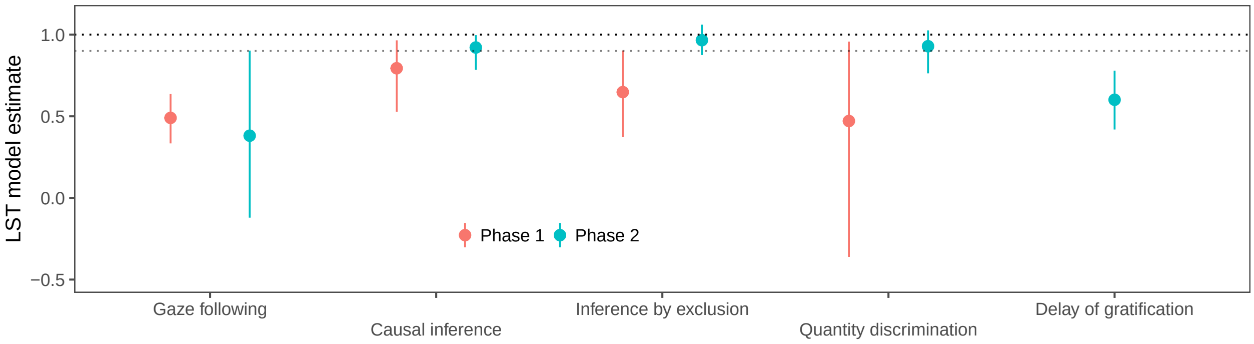

Supplementary Figure 33: Posterior means of auto-regressive β coefficients for LST-AR models with 95% CrI based on data from N = 43 participants.

Simulations

Simulation setup

The purpose of the simulations was to check whether the results of the present study can be trusted given the models and the sample size. The results thus only apply to this specific case and (Bayesian) estimation process and do not constitute general recommendations to be used during study planning in the future.

Data were generated and estimated using MPlus 8.4. Data-generating values are based on the real-data application of the models to the available subset of the data at the time of conducting the simulation study. That is, data were simulated for 40 individuals (N) observed across 9 or 12 measurement occasions, with 5 or 7 observed categories per indicator. 1000 replications were simulated. Data estimation took place using the MPlus default priors. In case of LST models for one construct, default priors were compared with IG(0.001, 0.001) priors set on all variance parameters (model did not include latent covariances). Two chains with a minimum of 5,000 iterations per chain and a thinning factor of 10 was applied (i.e. at last 50,000 iterations of which only every 10th was used for constructing the posterior distribution). Convergence was assumed and estimation stopped when the PSR fell below 1.05 for the first time after the minimum number of iterations was reached.

Simulation results

In the following, the 95% coverage rate, the Relative Parameter Estimation Bias (deviation between average estimate and population parameter divided by the population parameter), the Mean Squared Error, absolute bias, as well as Relative Standard Error Bias are displayed for every simulated model (Supplementary Figure 34 - 42). Relative parameter and standard error biases below 0.1 (that is $<$ 10%) are considered acceptable.

Parameters in the latent state models for one construct are estimated accurately, with relative biases below a cutoff of 10% bias and good coverage rates, irrespective of simulating 7 or 5 observed ordered categories for the observed indicators.

Latent state-trait models for one construct with latent state residual variances fixed across time show good estimation performance, with both default or adapted inverse gamma priors. When freely estimating latent state residual variances across time points (i.e., no restrictions on variances), model parameters are not estimated accurately under the simulated sample sizes, irrespective of the prior choice.

Latent state-trait models for a combination of two constructs with latent variances and covariances of the state residual variances restricted to equality across time points work well. Models with freely estimated variances and covariances do not show good estimation performance. The same holds for the latent state model with two constructs and freely estimated variances and covariances.

In conclusion, latent state models for one construct (freely estimated variances) as well as latent state-trait models for one or two constructs with state residual variances restricted across time exhibit good estimation performance (low biases, high coverage) and application under the simulated samples size should be feasible in practice.

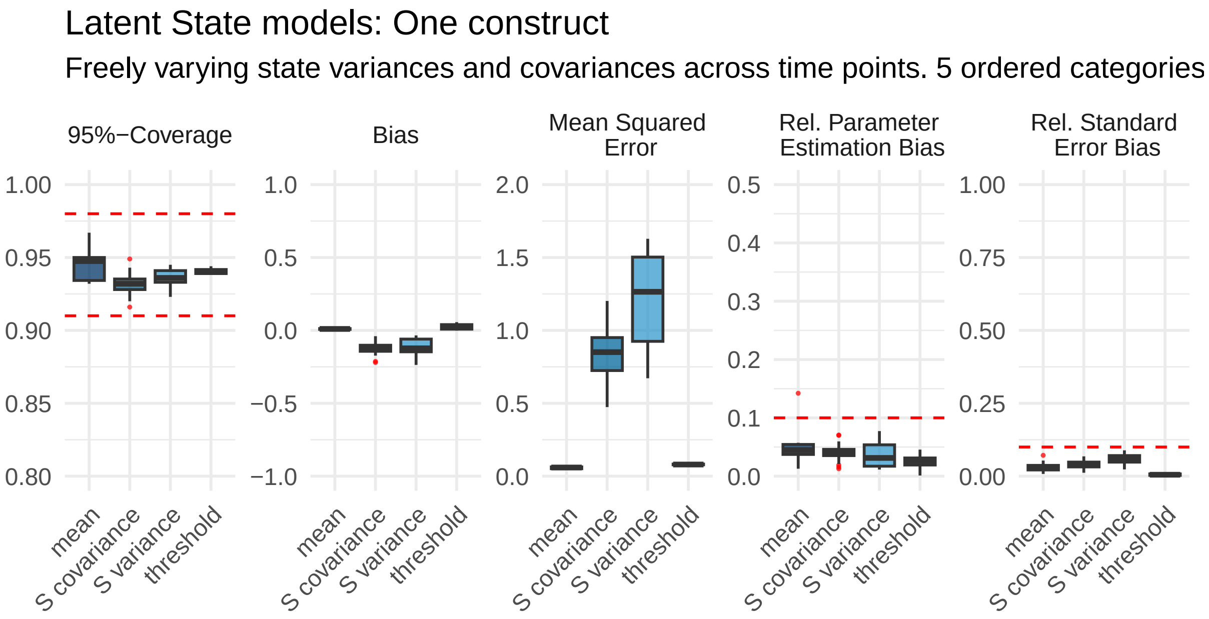

Supplementary Figure 34: Results of the simulation study for the latent state (LS) model including one construct with freely estimated latent state variances and covariances, spanning 9 measurement time points. Simulated sample size was N = 40. Ordinal indicators were simulated with 5 ordered categories. Boxplots display the distribution of the respective statistic across different parameters of the same parameter type. Whiskers show the minimum and the maximum. Red dots show ouliers. The box ranges from the 25% to the 75% quantile. Black line shows the median.

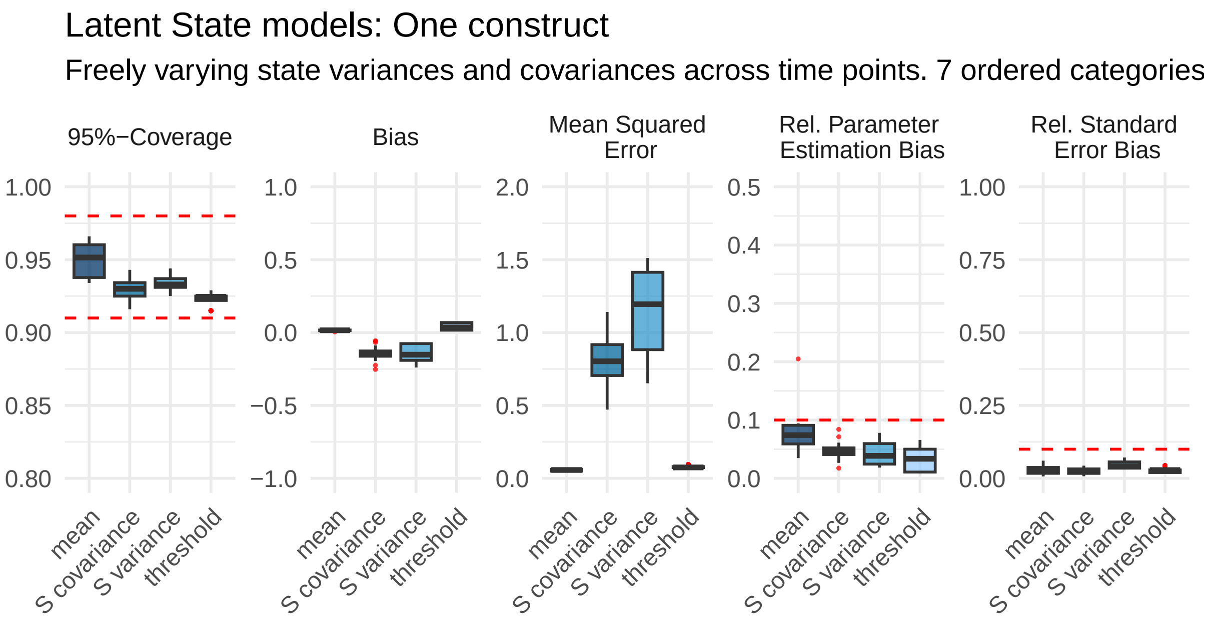

Supplementary Figure 35: Results of the simulation study for the latent state (LS) model including one construct with freely estimated latent atate variances and covariances, spanning 9 measurement time points. Simulated sample size was N = 40. Ordinal indicators were simulated with 7 ordered categories. Boxplots display the distribution of the respective statistic across different parameters of the same parameter type. Whiskers show the minimum and the maximum. Red dots show ouliers. The box ranges from the 25% to the 75% quantile. Black line shows the median.

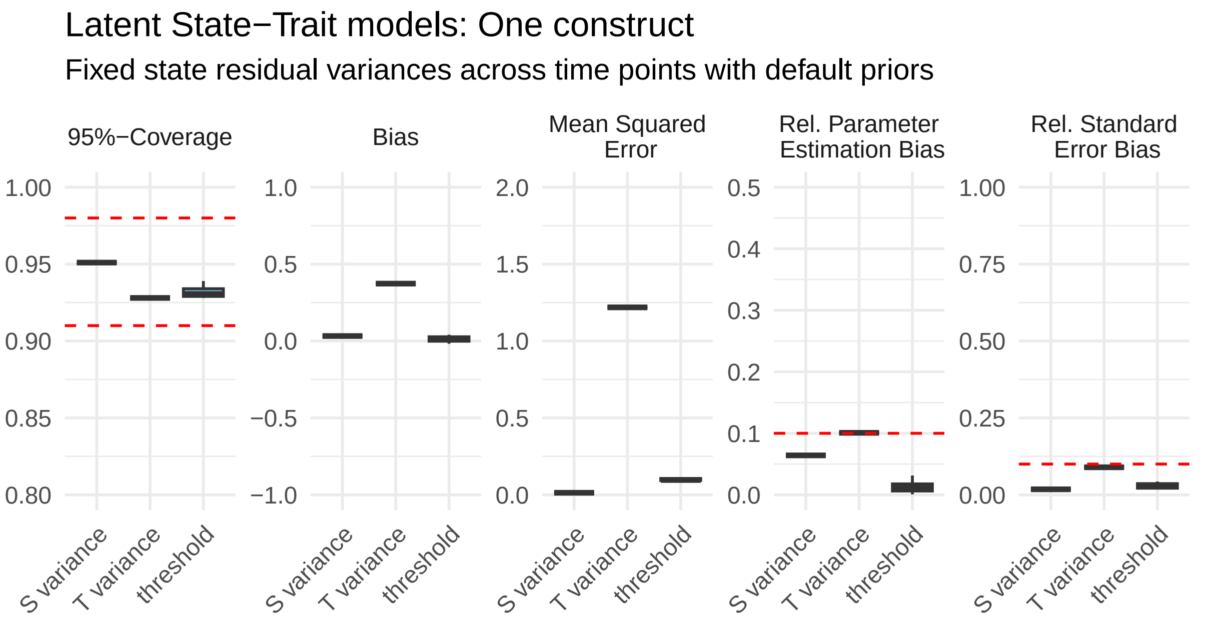

Supplementary Figure 36: Results of the simulation study for the latent state-trait (LST) model including one construct with latent state residual variances fixed to be equal across time, spanning 9 measurement time points. Simulated sample size was N = 40. MPlus default priors. Ordinal indicators were simulated with 7 ordered categories. Boxplots display the distribution of the respective statistic across different parameters of the same parameter type. Whiskers show the minimum and the maximum. Red dots show ouliers. The box ranges from the 25% to the 75% quantile. Black line shows the median.

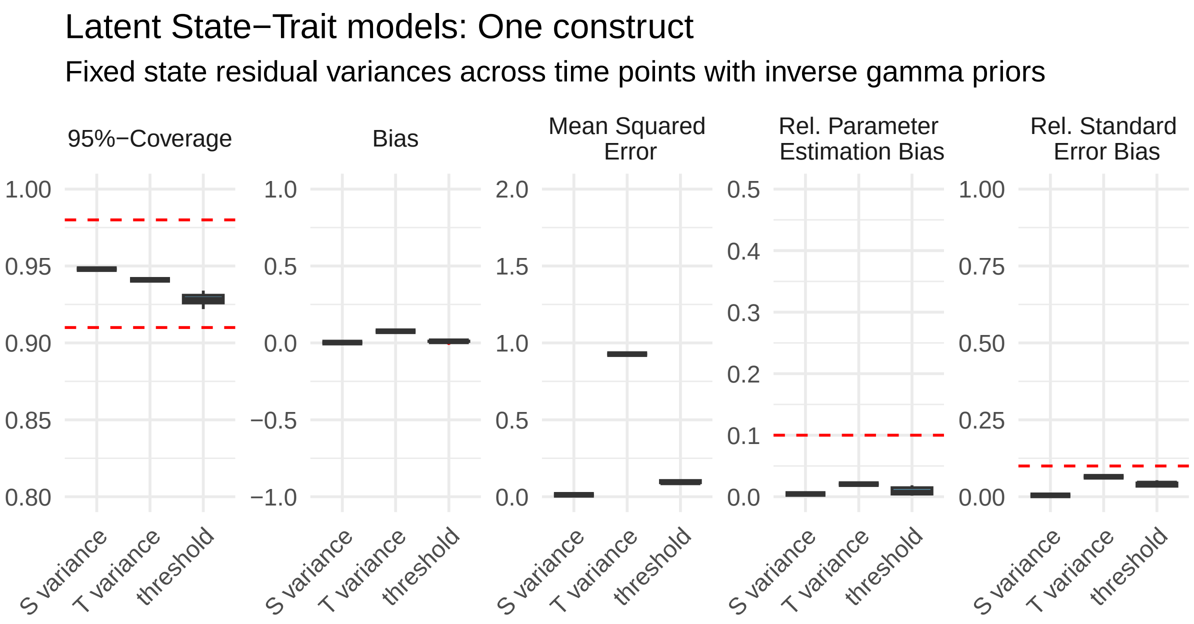

Supplementary Figure 37: Results of the simulation study for the latent state-trait (LST) model including one construct with latent state residual variances fixed to be equal across time, spanning 9 measurement time points. Simulated sample size was N = 40. Inverse gamma priors IG(0.001,0.001) for all variances. Ordinal indicators were simulated with 7 ordered categories. Boxplots display the distribution of the respective statistic across different parameters of the same parameter type. Whiskers show the minimum and the maximum. Red dots show ouliers. The box ranges from the 25% to the 75% quantile. Black line shows the median.

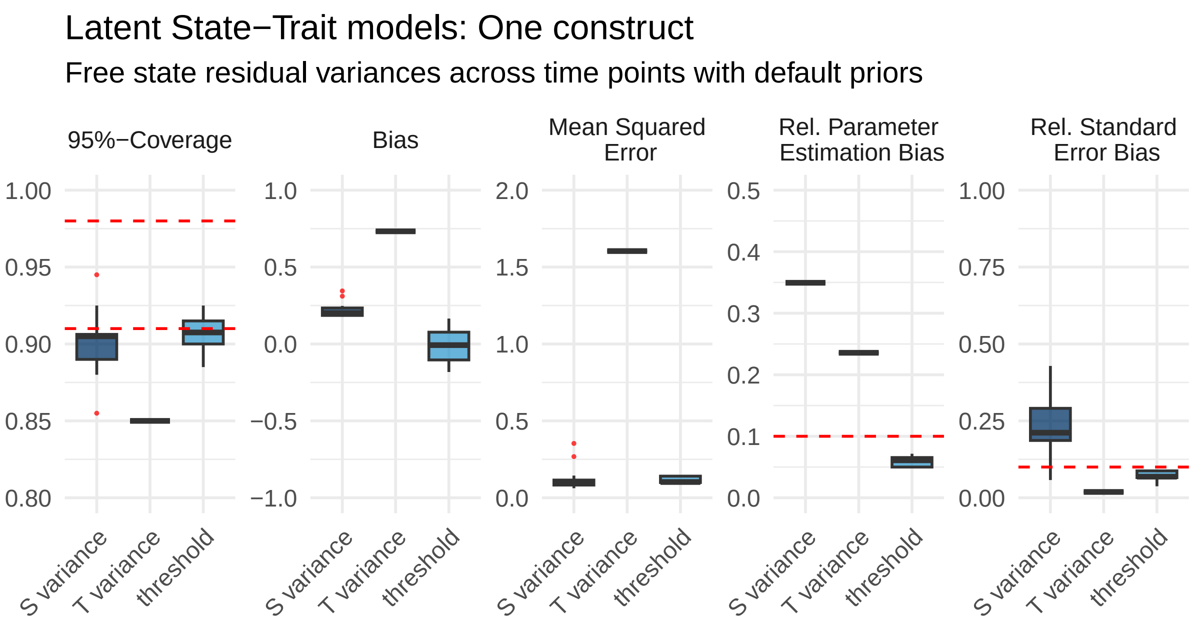

Supplementary Figure 38: Results of the simulation study for the latent state-trait (LST) model including one construct with latent state residual variances freely estimates across time, spanning 9 measurement time points. Simulated sample size was N = 40. Ordinal indicators were simulated with 7 ordered categories. Boxplots display the distribution of the respective statistic across different parameters of the same parameter type. Whiskers show the minimum and the maximum. Red dots show ouliers. The box ranges from the 25% to the 75% quantile. Black line shows the median.

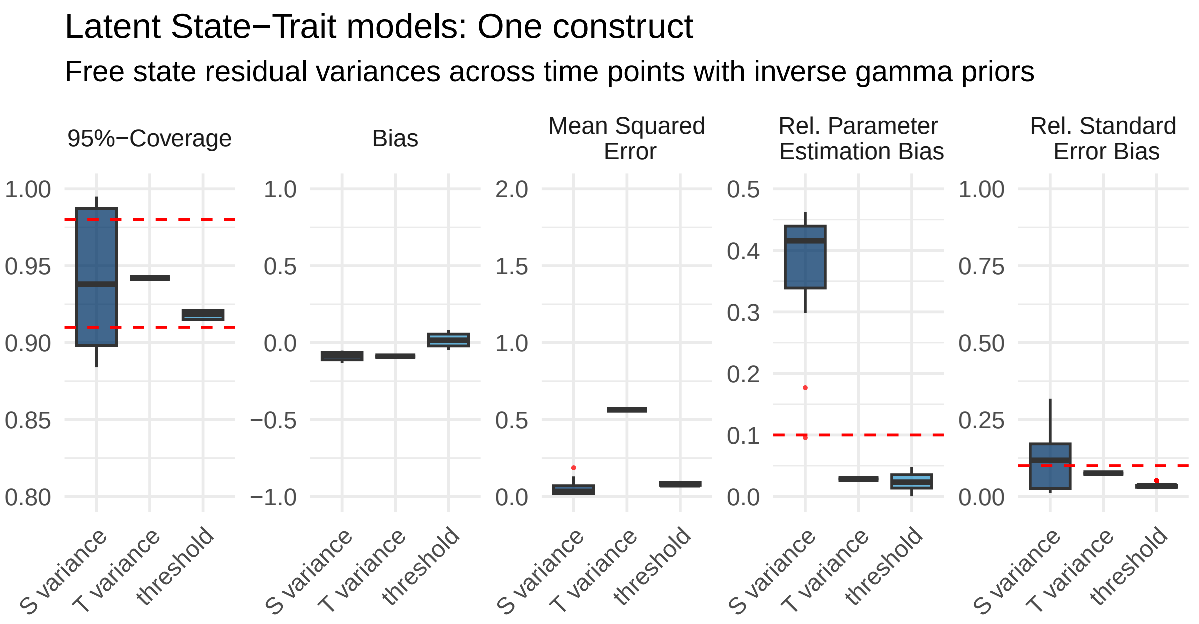

Supplementary Figure 39: Results of the simulation study for the latent state-trait (LST) model including one construct with latent state residual variances freely estimates across time, spanning 9 measurement time points. Simulated sample size was N = 40. Inverse gamma priors IG(0.001,0.001) for all variances. Ordinal indicators were simulated with 7 ordered categories. Boxplots display the distribution of the respective statistic across different parameters of the same parameter type. Whiskers show the minimum and the maximum. Red dots show ouliers. The box ranges from the 25% to the 75% quantile. Black line shows the median.

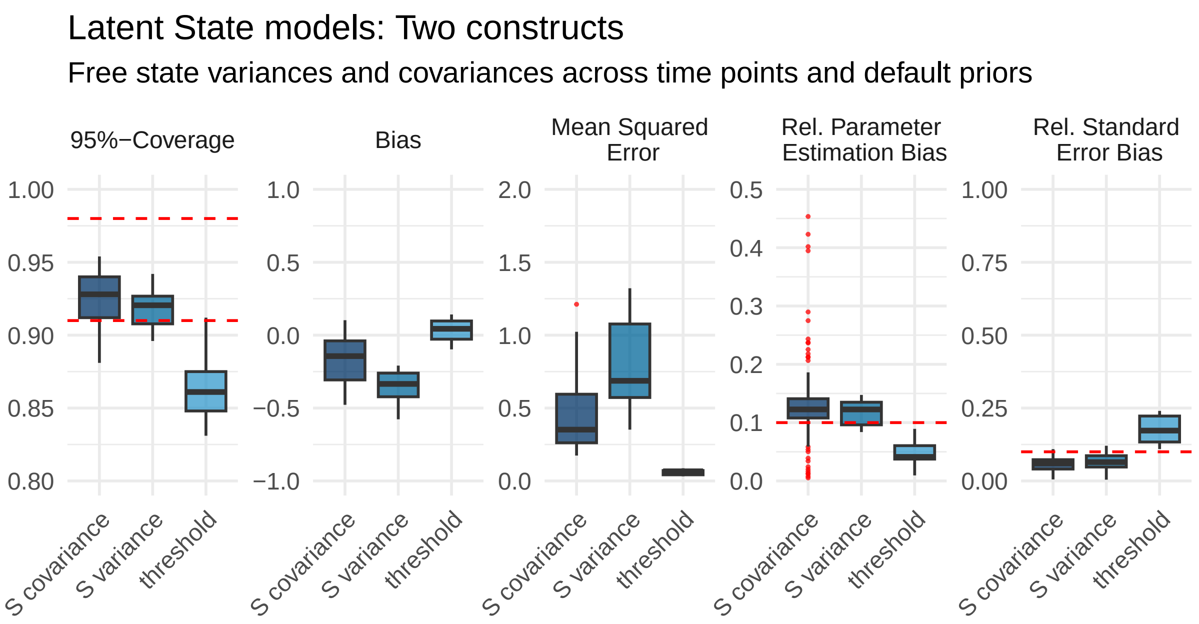

Supplementary Figure 40: Results of the simulation study for the latent state model including two constructs with latent state variances freely estimates across time, spanning 9 measurement time points. Simulated sample size was N = 40. Ordinal indicators were simulated with 7 ordered categories. Boxplots display the distribution of the respective statistic across different parameters of the same parameter type. Whiskers show the minimum and the maximum. Red dots show ouliers. The box ranges from the 25% to the 75% quantile. Black line shows the median.

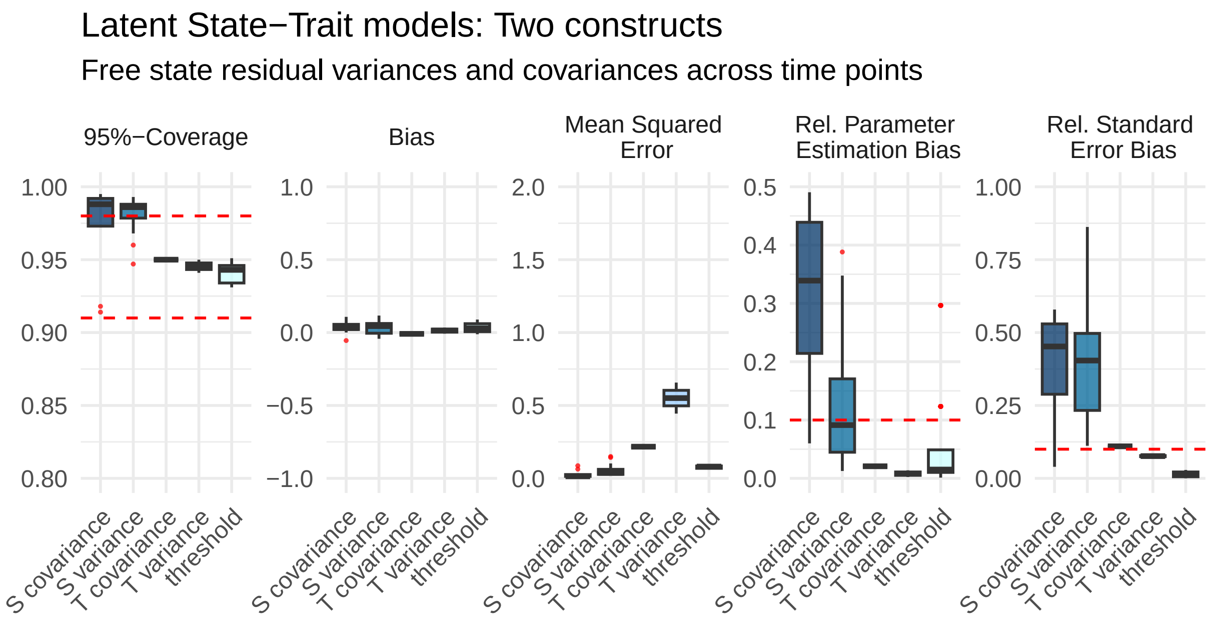

Supplementary Figure 41: Results of the simulation study for the latent state-trait (LST) model including two constructs with free latent state residual variances across time, spanning 9 measurement time points. Simulated sample size was N = 40. Ordinal indicators were simulated with 7 ordered categories. Boxplots display the distribution of the respective statistic across different parameters of the same parameter type. Whiskers show the minimum and the maximum. Red dots show ouliers. The box ranges from the 25% to the 75% quantile. Black line shows the median.

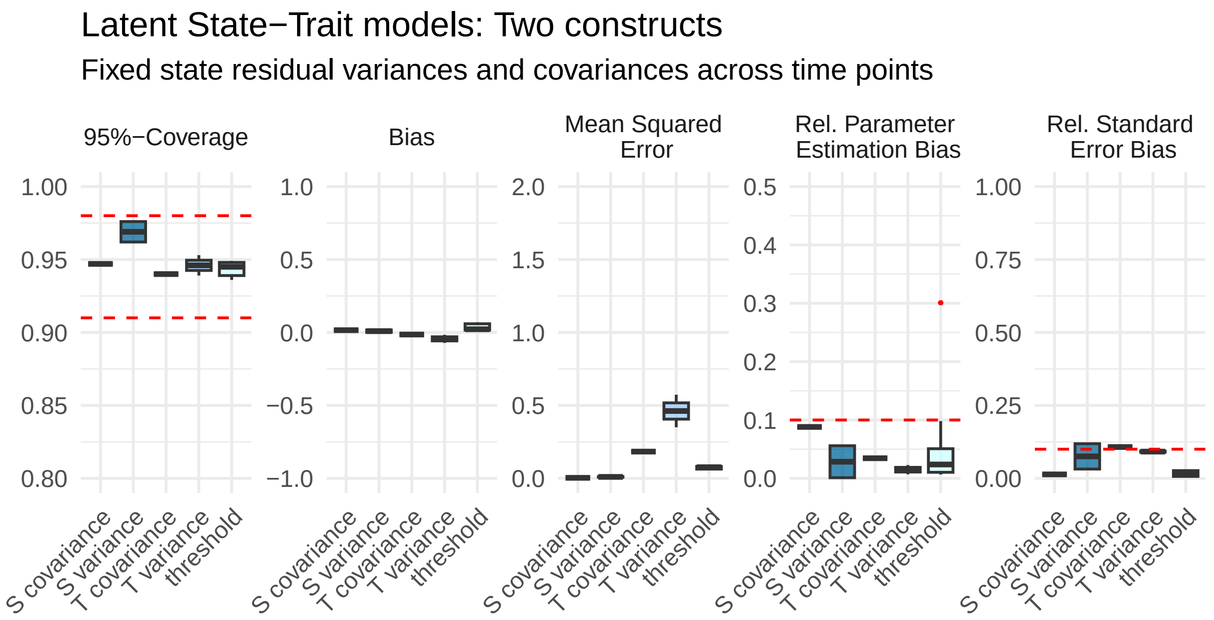

Supplementary Figure 42: Results of the simulation study for the latent state-trait (LST) model including two constructs with fixed latent state residual variances across time, spanning 9 measurement time points. Simulated sample size was N = 40. Ordinal indicators were simulated with 7 ordered categories. Boxplots display the distribution of the respective statistic across different parameters of the same parameter type. Whiskers show the minimum and the maximum. Red dots show ouliers. The box ranges from the 25% to the 75% quantile. Black line shows the median.

Supplementary References

Asparouhov, T., & Muthén, B. (2010). Bayesian analysis in mplus: Technical implementation. Mplus technical report, Version 3. Retrieved from http://www.statmodel.com/download/Bayes3.pdf

Bollen, K. A. (1989). *Structural equations with latent variables* (Vol. 210). John Wiley & Sons.

Bräuer, J., Call, J., & Tomasello, M. (2005). All great ape species follow gaze to distant locations and around barriers. *Journal of Comparative Psychology*, *119*(2), 145.

Call, J. (2004). Inferences about the location of food in the great apes (pan paniscus, pan troglodytes, gorilla gorilla, and pongo pygmaeus). *Journal of Comparative Psychology*, *118*(2), 232.

Catalina, A., Bürkner, P.-C., & Vehtari, A. (2020). Projection predictive inference for generalized linear and additive multilevel models. *arXiv Preprint arXiv:2010.06994*.

Eid, M., Holtmann, J., Santangelo, P., & Ebner-Priemer, U. (2017). On the definition of latent-state-trait models with autoregressive effects: Insights from LST-r theory. *European Journal of Psychological Assessment*, *33*(4), 285.

Eid, M., & Kutscher, T. (2014). Statistical models for analyzing stability and change in happiness. In K. Sheldon & R. Lucas (Eds.), *Stability of happiness: Theories and evidence on whether happiness can change* (pp. 261–297). Elsevier.

Geiser, C. (2020). *Longitudinal structural equation modeling with mplus: A latent state-trait perspective.* Guilford Publications.

Hanus, D., & Call, J. (2007). Discrete quantity judgments in the great apes (pan paniscus, pan troglodytes, gorilla gorilla, pongo pygmaeus): The effect of presenting whole sets versus item-by-item. *Journal of Comparative Psychology*, *121*(3), 241.

Haun, D. B., Call, J., Janzen, G., & Levinson, S. C. (2006). Evolutionary psychology of spatial representations in the hominidae. *Current Biology*, *16*(17), 1736–1740.

Hoyle, R. H. (2012). *Handbook of structural equation modeling.* Guilford press.

Leckie, G. (2019). Multiple membership multilevel models. Retrieved from https://arxiv.org/abs/1907.04148

Lüdtke, O., Robitzsch, A., & Wagner, J. (2018). More stable estimation of the STARTS model: A bayesian approach using markov chain monte carlo techniques. *Psychological Methods*, *23*(3), 570.

Meredith, W. (1993). Measurement equivalence, factor analysis and factorial equivalence. *Psychometrika*, *58*(4), 525–543.

Millsap, R. E., & Yun-Tein, J. (2004). Assessing factorial invariance in ordered-categorical measures. *Multivariate Behavioral Research*, *39*(3), 479–515.

Muthén, B. (1984). A general structural equation model with dichotomous, ordered categorical, and continuous latent variable indicators. *Psychometrika*, *49*(1), 115–132.

Muthén, L. K., & Muthén, B. (1998-2017). *Mplus user's guide. Eighth edition.* Los Angeles, CA:Muthén & Muthén.

Pavone, F., Piironen, J., Bürkner, P.-C., & Vehtari, A. (2020). Using reference models in variable selection. Retrieved from https://arxiv.org/abs/2004.13118

Piironen, J., Paasiniemi, M., Catalina, A., Weber, F., & Vehtari, A. (2022). projpred: Projection predictive feature selection. Retrieved from https://mc-stan.org/projpred/

Piironen, J., Paasiniemi, M., & Vehtari, A. (2020). Projective inference in high-dimensional problems: Prediction and feature selection. *Electronic Journal of Statistics*, *14*(1), 2155–2197. http://doi.org/10.1214/20-EJS1711

Piironen, J., & Vehtari, A. (2017). Comparison of bayesian predictive methods for model selection. *Statistics and Computing*, *27*, 711–735. http://doi.org/10.1007/s11222-016-9649-y

Rosati, A. G., Stevens, J. R., Hare, B., & Hauser, M. D. (2007). The evolutionary origins of human patience: Temporal preferences in chimpanzees, bonobos, and human adults. *Current Biology*, *17*(19), 1663–1668.

Samejima, F. (1969). Estimation of latent ability using a response pattern of graded scores. *Psychometrika*, (34), 1–97.

Samejima, F. (1996). The graded response model. In W. van der Linden & R. Hambleton (Eds.), *Handbook of modern item response theory* (pp. 85–100). New York: Springer.

Snijders, T. A., & Kenny, D. A. (1999). The social relations model for family data: A multilevel approach. *Personal Relationships*, *6*(4), 471–486.

Steyer, R., Ferring, D., & Schmitt, M. J. (1992). States and traits in psychological assessment. *European*

Journal of Psychological Assessment, *8*, 79–98.
Steyer, R., Mayer, A., Geiser, C., & Cole, D. A. (2015). A theory of states and traits—revised. *Annual Review of Clinical Psychology*, *11*, 71–98.
Takane, Y., & De Leeuw, J. (1987). On the relationship between item response theory and factor analysis of discretized variables. *Psychometrika*, *52*(3), 393–408.
Uher, J. (2011). Individual behavioral phenotypes: An integrative meta-theoretical framework. Why "behavioral syndromes" are not analogs of "personality." *Developmental Psychobiology*, *53*(6), 521–548.
Wark, J. D., Cronin, K. A., Niemann, T., Shender, M., Horrigan, A., Kao, A., & Ross, M. R. (2019). Monitoring the behavior and habitat use of animals to enhance welfare using the ZooMonitor app. *Animal Behavior and Cognition*, *6*, 158–167.
