# Supplementary material for: Great ape cognition is structured by stable cognitive abilities and predicted by developmental conditions
Source: Nat Ecol Evol. 2023 Apr 27;7(6):927–38. doi: 10.1038/s41559-023-02050-8 (PMC10250201; doi:10.1038/s41559-023-02050-8)
Supplement: Supplementary file 2 — Reporting Summary [file 41559_2023_2050_MOESM2_ESM.pdf]

nature portfolio

Corresponding author(s): Manuel Bohn

Last updated by author(s): Aug 26, 2022

Reporting Summary

Nature Portfolio wishes to improve the reproducibility of the work that we publish. This form provides structure for consistency and transparency in reporting. For further information on Nature Portfolio policies, see our Editorial Policies and the Editorial Policy Checklist.

Statistics

For all statistical analyses, confirm that the following items are present in the figure legend, table legend, main text, or Methods section.

n/a	Confirmed	
☐	☒	The exact sample size (*n*) for each experimental group/condition, given as a discrete number and unit of measurement
☐	☒	A statement on whether measurements were taken from distinct samples or whether the same sample was measured repeatedly
☒	☐	The statistical test(s) used AND whether they are one- or two-sided<br>*Only common tests should be described solely by name; describe more complex techniques in the Methods section.*
☐	☒	A description of all covariates tested
☐	☒	A description of any assumptions or corrections, such as tests of normality and adjustment for multiple comparisons
☐	☒	A full description of the statistical parameters including central tendency (e.g. means) or other basic estimates (e.g. regression coefficient) AND variation (e.g. standard deviation) or associated estimates of uncertainty (e.g. confidence intervals)
☒	☐	For null hypothesis testing, the test statistic (e.g. *F*, *t*, *r*) with confidence intervals, effect sizes, degrees of freedom and *P* value noted<br>*Give P values as exact values whenever suitable.*
☐	☒	For Bayesian analysis, information on the choice of priors and Markov chain Monte Carlo settings
☐	☒	For hierarchical and complex designs, identification of the appropriate level for tests and full reporting of outcomes
☐	☒	Estimates of effect sizes (e.g. Cohen's *d*, Pearson's *r*), indicating how they were calculated

Our web collection on statistics for biologists contains articles on many of the points above.

Software and code

Policy information about availability of computer code

Data collection	No software was used to collect the data
Data analysis	R version 4.2; R packages used for inferential statistics: brms version 2.17; projpred version 2.1.2; Structural Equation Models were implemented in MPlus version 8.4.

For manuscripts utilizing custom algorithms or software that are central to the research but not yet described in published literature, software must be made available to editors and reviewers. We strongly encourage code deposition in a community repository (e.g. GitHub). See the Nature Portfolio guidelines for submitting code & software for further information.

Data

Policy information about availability of data

All manuscripts must include a data availability statement. This statement should provide the following information, where applicable:

- Accession codes, unique identifiers, or web links for publicly available datasets
- A description of any restrictions on data availability
- For clinical datasets or third party data, please ensure that the statement adheres to our policy

All data and analysis scripts can be found in the associated online repository (https://github.com/ccp-eva/laac).

Human research participants

Policy information about studies involving human research participants and Sex and Gender in Research.

Reporting on sex and gender	*Use the terms sex (biological attribute) and gender (shaped by social and cultural circumstances) carefully in order to avoid confusing both terms. Indicate if findings apply to only one sex or gender; describe whether sex and gender were considered in study design whether sex and/or gender was determined based on self-reporting or assigned and methods used. Provide in the source data disaggregated sex and gender data where this information has been collected, and consent has been obtained for sharing of individual-level data; provide overall numbers in this Reporting Summary. Please state if this information has not been collected. Report sex- and gender-based analyses where performed, justify reasons for lack of sex- and gender-based analysis.*
Population characteristics	*Describe the covariate-relevant population characteristics of the human research participants (e.g. age, genotypic information, past and current diagnosis and treatment categories). If you filled out the behavioural & social sciences study design questions and have nothing to add here, write "See above."*
Recruitment	*Describe how participants were recruited. Outline any potential self-selection bias or other biases that may be present and how these are likely to impact results.*
Ethics oversight	*Identify the organization(s) that approved the study protocol.*

Note that full information on the approval of the study protocol must also be provided in the manuscript.

Field-specific reporting

Please select the one below that is the best fit for your research. If you are not sure, read the appropriate sections before making your selection.

☐ Life sciences ☒ Behavioural & social sciences ☐ Ecological, evolutionary & environmental sciences

For a reference copy of the document with all sections, see nature.com/documents/nr-reporting-summary-flat.pdf

Behavioural & social sciences study design

All studies must disclose on these points even when the disclosure is negative.

Study description	Quantitative data obtained from behavioral experiments
Research sample	A total of 43 great apes participated at least once in one of the tasks. This included 8 Bonobos (3 females, age 7.30 to 39), 24 Chimpanzees (18 females, age 2.60 to 55.90), 6 Gorillas (4 females, age 2.70 to 22.60), and 5 Orangutans (4 females, age 17 to 41.20). Apes were housed at the Wolfgang Köhler Primate Research Center located in Zoo Leipzig, Germany.
Sampling strategy	We included all great apes living at Leipzig Zoo who were willing to participate in research.
Data collection	Data was collected via non-invasive behavioral experiments using materials such as cups and plates. A human experimenter presented the materials and recorded the responses on a coding sheet. In addition, data collection was video recorded and a second coder coded a subset of trials for inter-rater reliability. The experimenter was not blind to the conditions.<br>Participation was voluntary, participants were not deprived of food or water and all food received during the experiments was given in addition to the regular daily diet.
Timing	Phase one lasted from August 1st, 2020 until March 5th, 2021; phase 2 lasted from May 26th, 2021 to December 4th, 2021
Data exclusions	No data was excluded
Non-participation	This was a longitudinal study and not all participants from the above mentioned pool participated at all time points. The sample size at each time point ranged from 22 to 43 individuals.
Randomization	This was an individual differences study, thus, all participants received the tasks in the same order.

Reporting for specific materials, systems and methods

We require information from authors about some types of materials, experimental systems and methods used in many studies. Here, indicate whether each material, system or method listed is relevant to your study. If you are not sure if a list item applies to your research, read the appropriate section before selecting a response.

Materials & experimental systems

n/a	Involved in the study	
☒	☐	Antibodies
☒	☐	Eukaryotic cell lines
☒	☐	Palaeontology and archaeology
☐	☒	Animals and other organisms
☒	☐	Clinical data
☒	☐	Dual use research of concern

Methods

n/a	Involved in the study	
☒	☐	ChIP-seq
☒	☐	Flow cytometry
☒	☐	MRI-based neuroimaging

Animals and other research organisms

Policy information about studies involving animals; ARRIVE guidelines recommended for reporting animal research, and Sex and Gender in Research

Laboratory animals	bonobos (Pan paniscus), chimpanzees (Pan troglodytes), gorillas (Gorilla gorilla), orangutans (Pongo abelii).
Wild animals	The study did not involve wild animals
Reporting on sex	Sex was considered as a predictor variable used to explain individual differences and was determined based on external genitalia.
Field-collected samples	The study did not involve samples collected from the field
Ethics oversight	The study was approved by an internal ethics committee at the Max Planck Institute for Evolutionary Anthropology.

Note that full information on the approval of the study protocol must also be provided in the manuscript.
